# Supplementary material for: Antibiotics from Insect-Associated Actinobacteria
Source: Biology (Basel). 2022 Nov 18;11(11):1676. doi: 10.3390/biology11111676 (PMC9687666; doi:10.3390/biology11111676)
Supplement: Supplementary file 1 [file biology-11-01676-s001.zip › biology-2024921-supplementary.pdf]

Supplementary Information

(Schemes S1-S4, Tables S1-S2, Figure S1, Lists S1-S2)

Antibiotics from insect-associated actinobacteria

Anna A. Baranova[1,2], Yuliya V. Zakalyukina[3], Anna A. Ovcharenko[1,4], Vladimir A. Korshun[1], Anton P. Tyurin[1]

1. Shemyakin-Ovchinnikov Institute of Bioorganic Chemistry, Miklukho-Maklaya 16/10, 117997 Moscow, Russia
2. Gause Institute of New Antibiotics, Bol'shaya Pirogovskaya 11, 119021 Moscow, Russia
3. Department of Soil Science, Lomonosov Moscow State University, Leninskie Gory 1-12, 119991 Moscow, Russia
4. Mendeleev University of Chemical Technology of Russia, Miusskaya sq. 9, Moscow 125047, Russian Federation

CONTENTS:

Scheme S1. Peptide antibiotics **2**
Scheme S2. Polyketide antibiotics **4**
Scheme S3. Alkaloid antibiotics **7**
Scheme S4. Other antibiotics **8**
Table S1. Selected publications **9**
Table S2. Media used for actinobacteria isolation **20**
Figure S1. Media used for actinobacteria isolation **22**
List S1. References for tables S1 and S2 .... **23**
List S2. Excluded publications **29**

Scheme S1. Peptide antibiotics

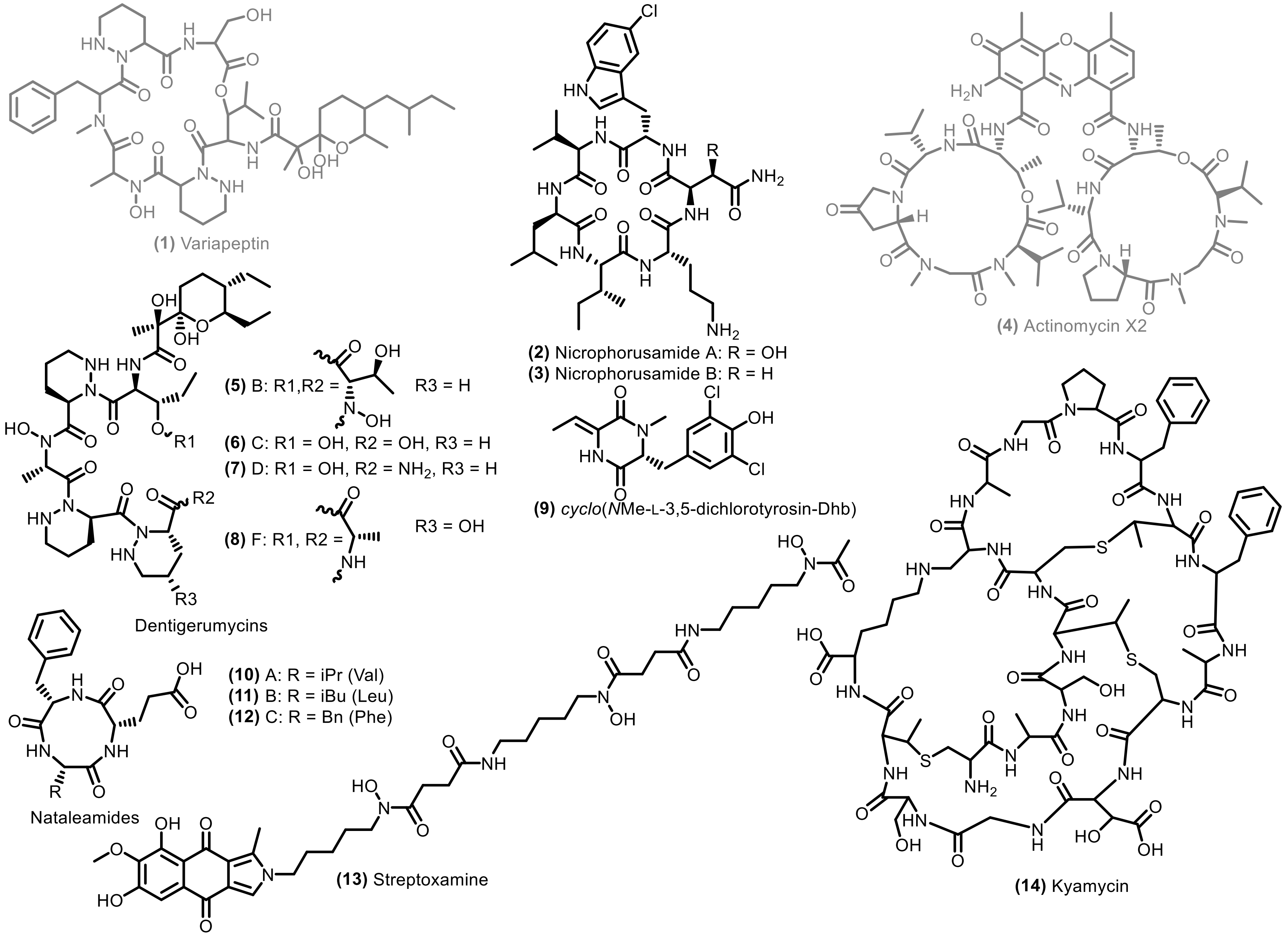

Back to contents

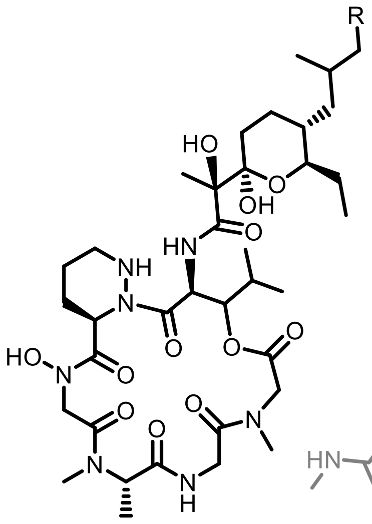

(15) Meliponamycin A: R = H
(16) Meliponamycin B: R = CH_3

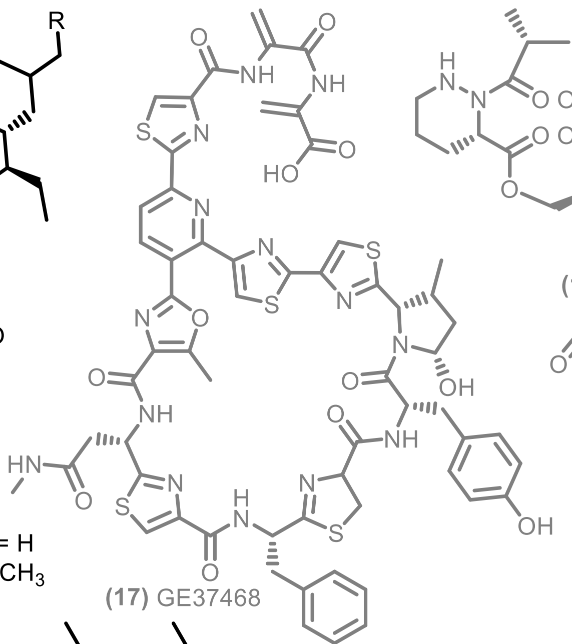

(17) GE37468

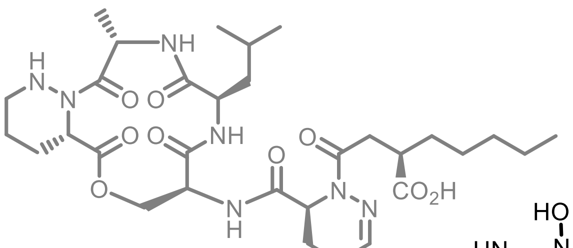

(18) Lydiamycin A

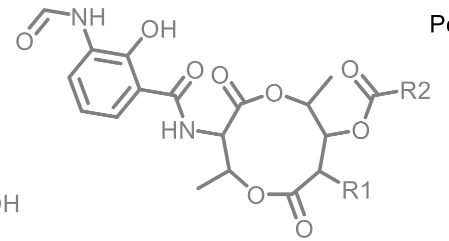

(19) Antimycin A complex
(R1,R2 - alkyls)

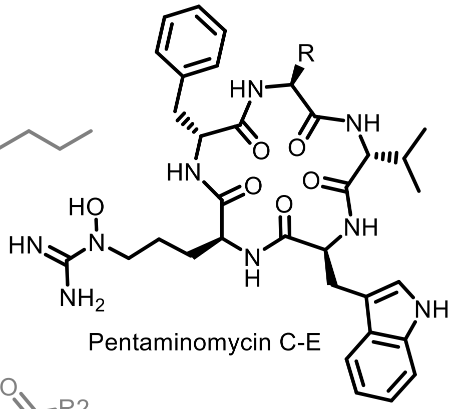

Pentaminomycin C-E
(20) C: R = *i*Bu (Leu)
(21) D: R = *i*Pr (Val)
(22) E: R = Bn (Phe)

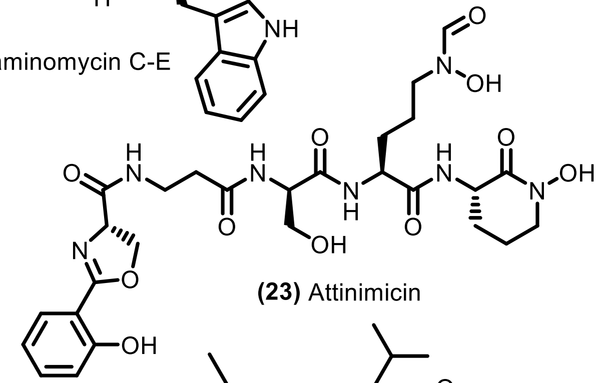

(23) Attinimicin

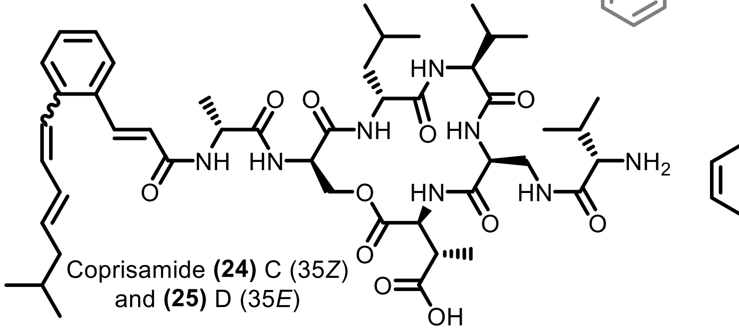

Coprisamide **(24)** C (35*Z*)
and **(25)** D (35*E*)

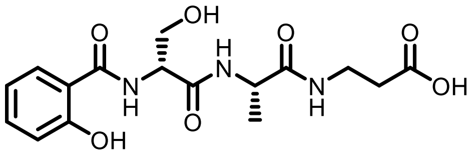

(26) Madurastatin G1

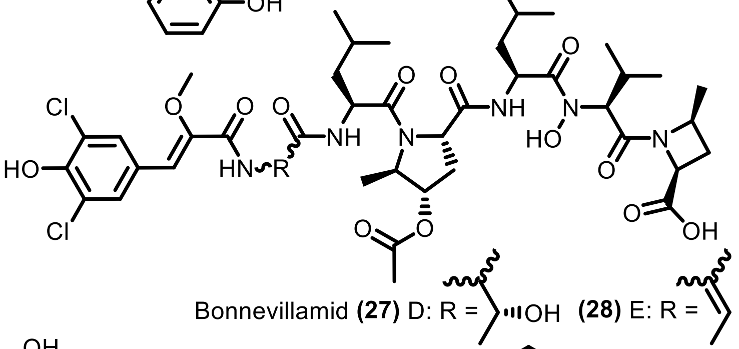

Bonnevillamid **(27)** D: R = ; **(28)** E: R =

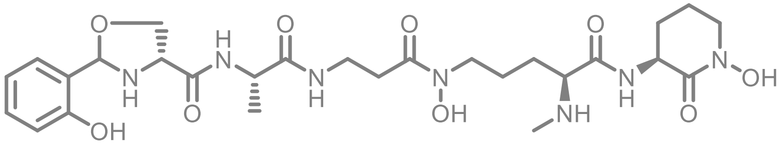

(29) Madurastatin A1

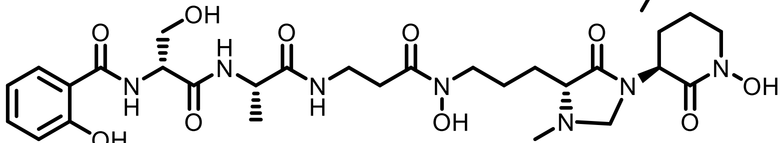

(31) Madurastatin E1

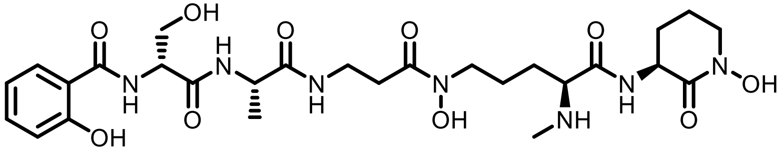

(30) Madurastatin A2

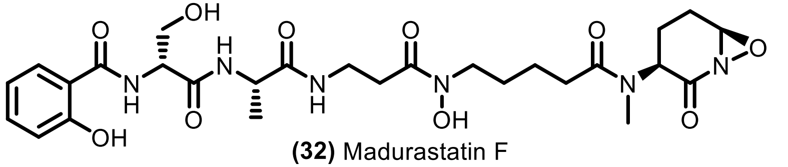

(32) Madurastatin F

Back to contents

SCHEME S2. POLYKETIDE ANTIBIOTICS

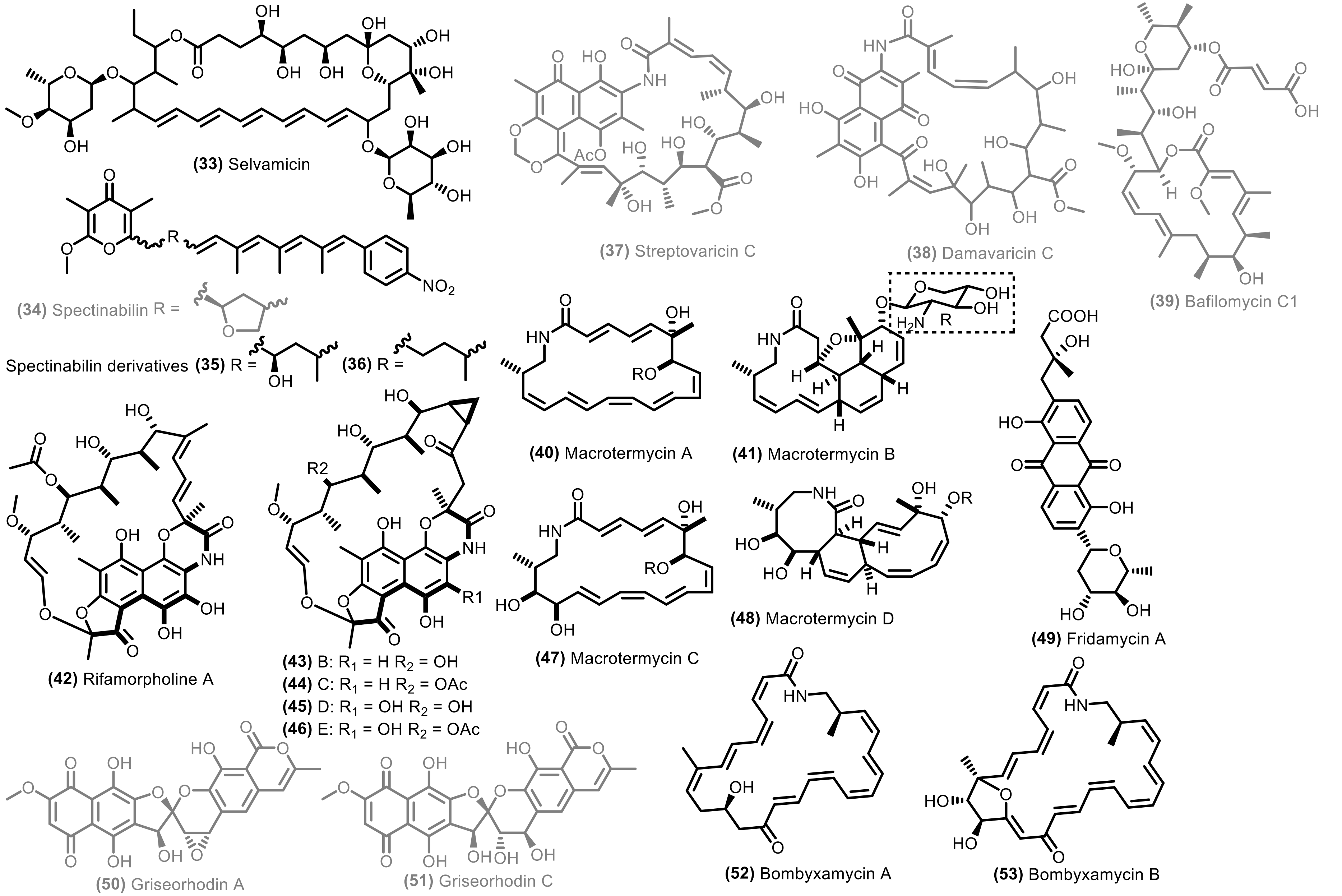

Back to contents

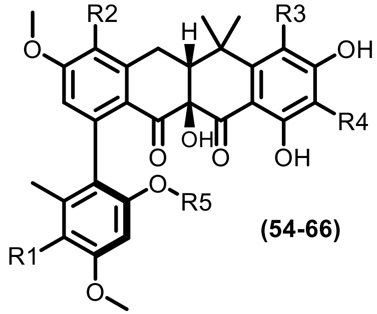

(54-66)

Formicamycin A R1 = H, R2 = Cl, R3 = H, R4 = H, R5 = CH_3
Formicamycin B R1 = Cl, R2 = Cl, R3 = H, R4 = H, R5 = H
Formicamycin C R1 = H, R2 = Cl, R3 = Cl, R4 = H, R5 = CH_3
Formicamycin D R1 = Cl, R2 = Cl, R3 = Cl, R4 = H, R5 = H
Formicamycin E R1 = Cl, R2 = Cl, R3 = Cl, R4 = H, R5 = CH_3
Formicamycin F R1 = Cl, R2 = Cl, R3 = H, R4 = Cl, R5 = CH_3
Formicamycin G R1 = H, R2 = Cl, R3 = H, R4 = Cl, R5 = CH_3
Formicamycin H R1 = Cl, R2 = H, R3 = Cl, R4 = Cl, R5 = CH_3
Formicamycin I R1 = Cl, R2 = Cl, R3 = Cl, R4 = Cl, R5 = H
Formicamycin J R1 = Cl, R2 = Cl, R3 = Cl, R4 = Cl, R5 = CH_3
Formicamycin K R1 = H, R2 = Cl, R3 = Br, R4 = Cl, R5 = CH_3
Formicamycin L R1 = Cl, R2 = Cl, R3 = Br, R4 = Cl, R5 = CH_3
Formicamycin M R1 = H, R2 = Br, R3 = H, R4 = H, R5 = CH_3

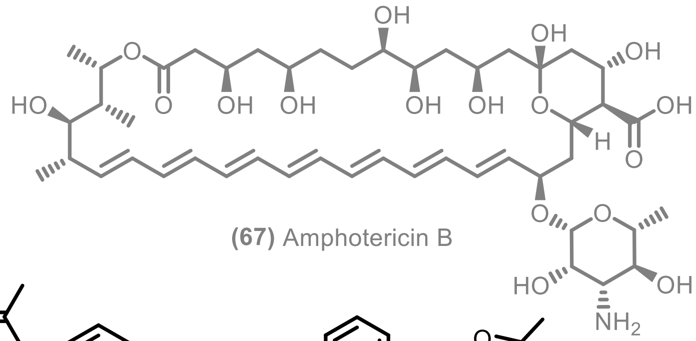

(67) Amphotericin B

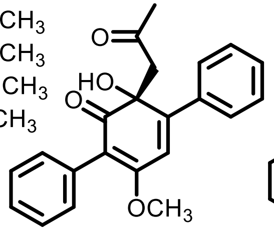

(71) Strepantibin A

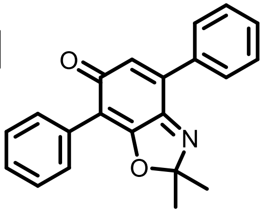

(72) Strepantibin B

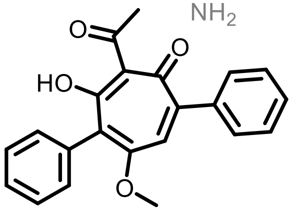

(73) Strepantibin C

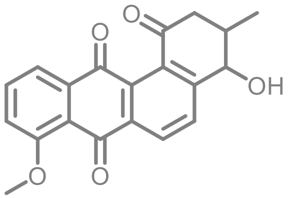

(68) Fugianmycin B

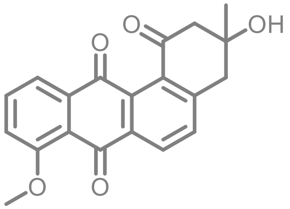

(69) 8-*O*-Methyltetrangomycin

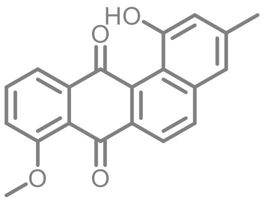

(70) 8-*O*-Methyltetrangulol

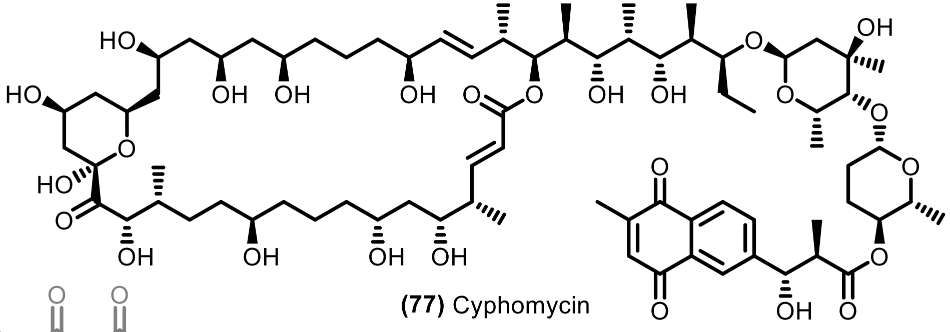

(77) Cyphomycin

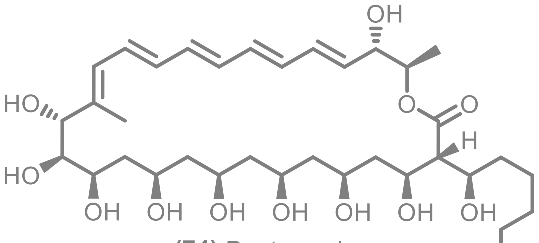

(74) Pentamycin

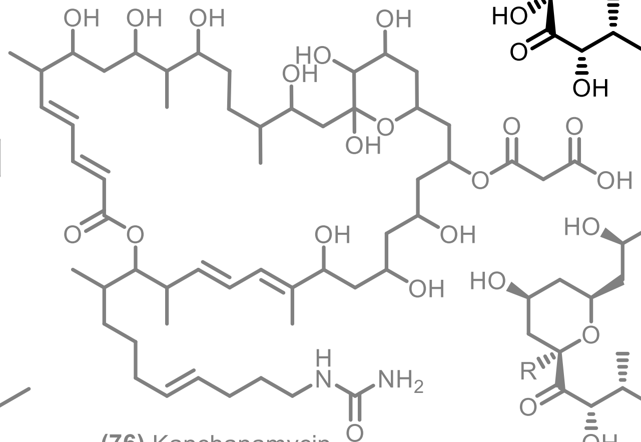

(76) Kanchanamycin

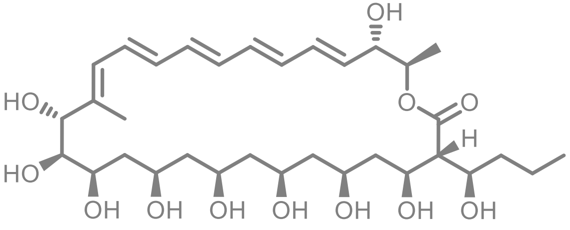

(75) 1',14-dihydroxyisochainin

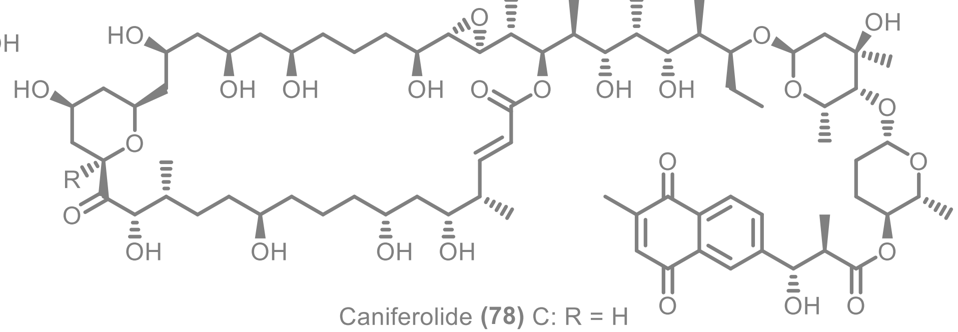

Caniferolide **(78)** C: R = H
(79) GT-35: R = OH

Back to contents

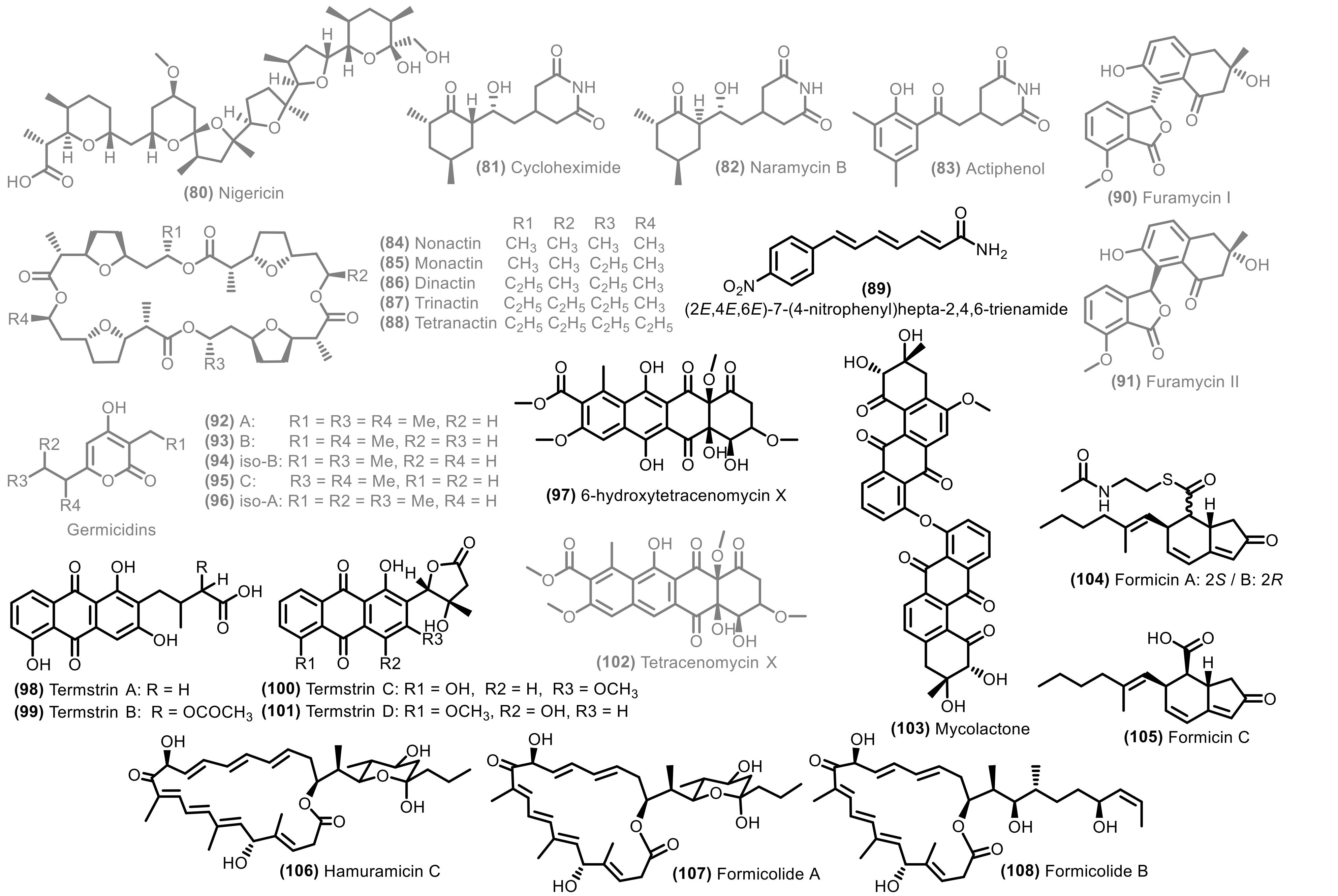

Back to contents

Scheme S3. Alkaloid antibiotics

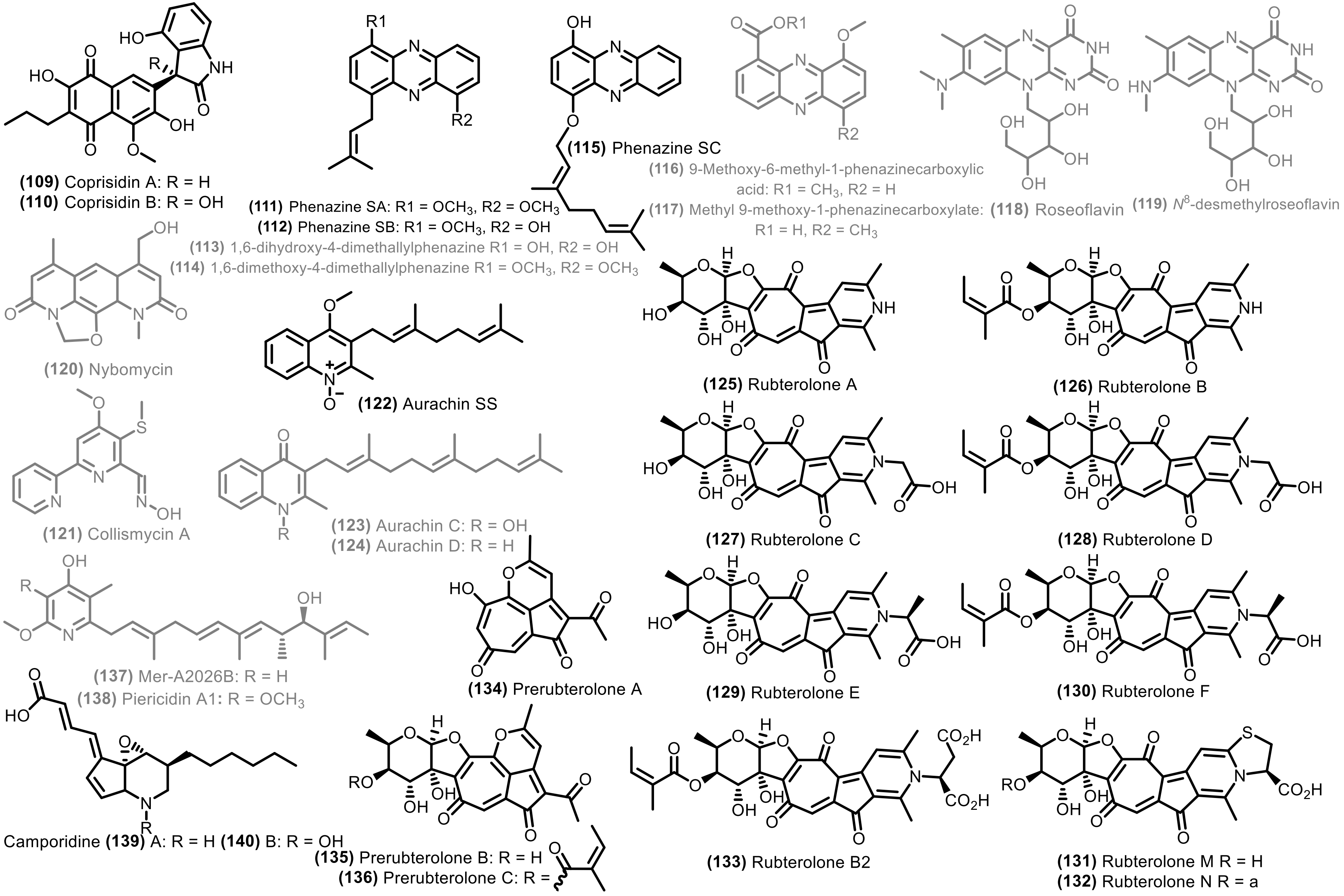

Back to contents

Scheme S4. Other antibiotics

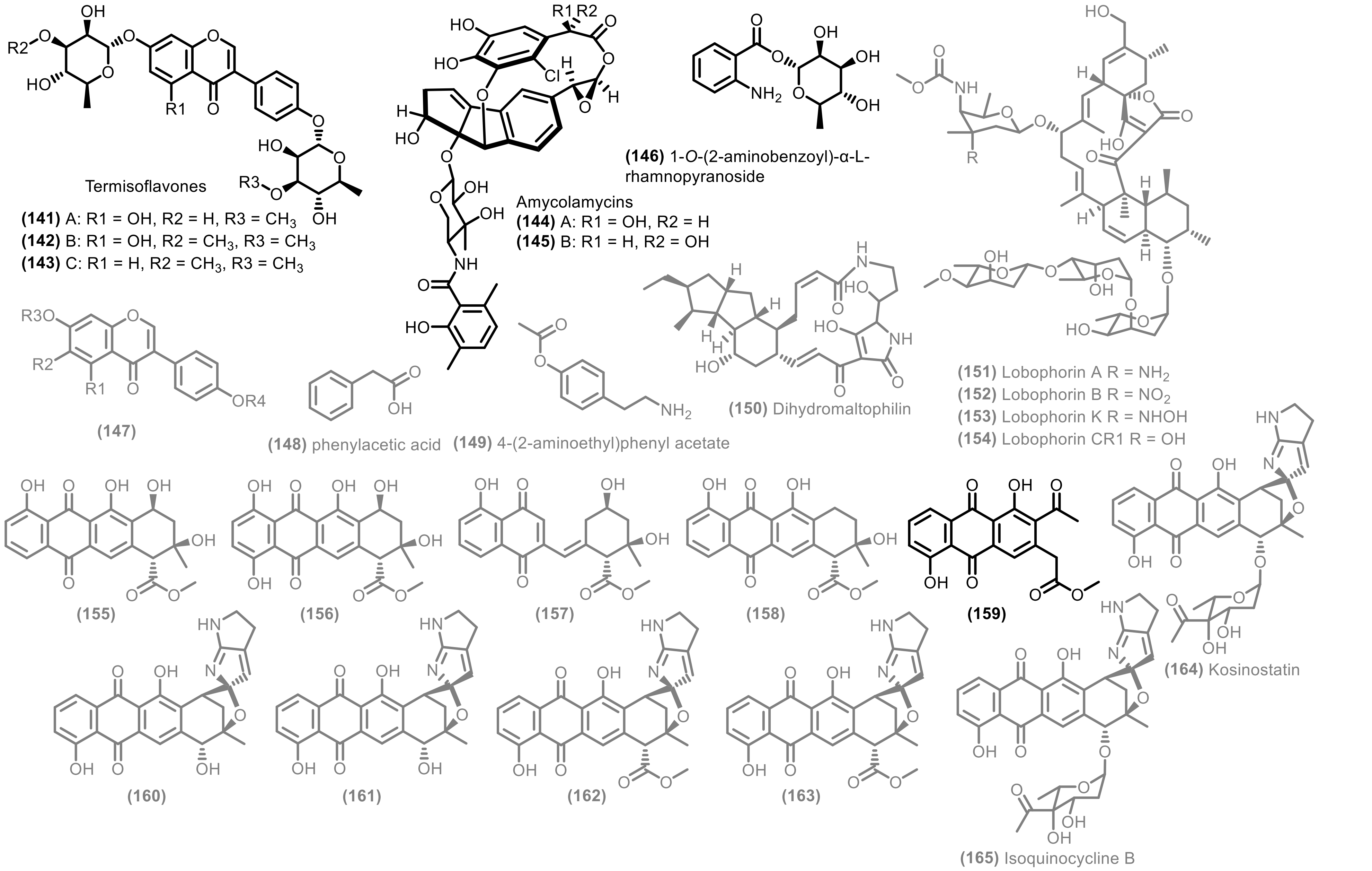

Back to contents

TABLE S1. SELECTED PUBLICATIONS

* See Table S2 for details

Structure	Actinobacteria	Insect host	Isolation medium*	Activity/Ecological role	Location	Reference
**(97), (102)**	*Amycolatopsis camponoti* A23	ant *Camponotus vagus* (worker)	AI (nystatin 250 mg, nalidixic acid 10 mg)	Tetracenomycin X and its new congener 6-hydroxytetracenomycin X have antimicrobial and cytotoxic activity.	Kasimovsky District (N 55.01138, E 41.73078), Ryazan region, Russia	[1]
**(106)**	*Streptomyces* sp. MBP16	wasp *Vespa crabro flavofasciata* (gut)	A1	Hamuramicin C demonstrated significant inhibitory activity against diverse human cancer cell lines (HCT116, A549, SNU-638, SK-HEP-1, and MDA-MB-231).	Gwanak Mountain, Seoul, Republic of Korea	[2]
no compounds	*Brachybacterium phenoliresistens* MO and *Microbacterium* sp.	ant *Messor orientalis*	G1 (160 mg/L cycloheximide, 50 mg/L of nalidixic acid)	Antifungal activity against *Peronophythora litchi*, *Rhizoctonia solani*, *Colletotrichum siamense*.	Wujiaqu, Xinjiang, China	[3]
no compounds	*Streptomyces* sp. W7	undefined termites	G1 (50 mg/L of bichromate)	*Streptomyces* sp. W7 had strong inhibitory activity against plant pathogenic fungi *C. musae*, *C. cassiicola*, *P. aphanidermatum*, with strong antibacterial persistence and antibacterial stability.	Tropical plants garden of Chinese Academy of Tropical Agricultural Sciences in Haikou, Hainan, China	[4]
**(26), (29-32)**	*Actinomadura* sp. RB99 (in co-culture with *Pseudoxylaria* sp. X802)	(previously described)	(previously described)	Madurastatins act as a siderophores.	(previously described)	[5]
no compounds	*Streptomyces* sp. ACZ2-27 and ASC3-2	bee *Apis andreniformis*	Cz and SCN (nalidixic acid and nystatin 25 mg)	Streptomycetes show antimicrobial activity against black rot pathogen (*Xanthomonas campestris* pv. *campestris*, by streak method).	The local villages in Mae-rim district, Chiang Mai province, Thailand	[6]
**NH_3** (ammonia)	*Streptomyces* sp. Av25_4	ant *Acromyrmex volcanus* (cuticle)	AI	*Streptomyces* sp. Av25_4 overproduces ammonia (up to 8 mM) which completely inhibited the growth of *E. weberi* due to its strong basic pH. Additionally, other symbionts from different *Acromyrmex* ants inhibited *E. weberi* by production of ammonia. The waste of ca. one third of	Gamboa, Panama	[7]

				Acomyrmex and *Atta* leaf-cutting ant colonies was strongly basic due to ammonia (up to ca. 8 mM) suggesting its role in nest hygiene. *Escovopsis* is sensitive to ammonia in contrast to the garden fungus *L. gongylophorus*.		
**(27, 28)**	*Streptomyces* sp. UTZ13	beetle *Nicrophorus concolor* (exoskeleton)	A1+C	Bonnevillamides D and E reversed the fibril formation by inducing the monomerization of amyloid-β aggregates (anti-Alzheimer's activity).	Maebong Mountain, Seoul, Republic of Korea	[8]
**(24, 25)**	*Micromonospora* sp. UTJ3	beetle *Silpha perforata* (gut)	PD	Coprisamides have weak activity against the *Mycobacterium tuberculosis* mc2 6230 strain and induce quinone reductase (QR) activity	Gwanak Mountain, Seoul, Republic of Korea	[9]
**(74, 75)**	*Streptomyces* sp. HF10	termite *Macrotermes barneyi* (foregut)	PY-CMC	Two pentene macrolides (pentamycin and 1,14- dihydroxyisochainin) were firstly purified from *Streptomyces* strain HF10, both exhibiting higher activity against *Xylaria* sp. and *M. anisopliae* than cultivar *Termitomyces*. These results indicate that polyene-producing *Streptomyces* from the guts of *M. barneyi* have strong inhibition to competitor fungus and polyenes contribute to inhibitory effects on *Xylaria* sp.	Hunan (N 26.5800, E 112.9600), China	[10]
**(103)**	*Amycolatopsis* sp. HCa1	grasshopper *Oxya chinensis* (gut)	no data	Mycolactone did not exhibited cytotoxic effects against the tested human carcinoma cell lines (cervical cancer cell line HeLa, gastric adenocarcinoma cell line SGC-7901 and lung adenocarcinoma cell line SPC-A-1) at concentration of 100 µM.	Nanchang, Jiangxi Province, China	[11]
**(77, 79)**	*Streptomyces* sp. ISID311	ant *Cyphomyrmex* sp.	Ch (40 mg nystatin, 50 mg cycloheximide)	Compounds displayed high antagonism against different strains of *Escovopsis* sp., pathogen fungi specialized to the fungus-growing ant system. Compounds also exhibited potent antiprotozoal activity against intracellular amastigotes of the human parasite *Leishmania donovani*.	campus of the University of São Paulo – Ribeirão Preto (S 21.167889,W 47.847083), Brazil	[12]
**(84-88)**	*Streptomyces* sp. M54	wasp *Polybia plebeja* (pupae)	Ch	Growth inhibition bioassays show that this bacterium produces antimicrobial	Las Brisas Nature Reserve, Limón	[13]

				compounds that are active against *Hirsutella citriformis*, a natural fungal enemy of its host, and the human pathogens *Staphylococcus aureus* and *Candida albicans*. Analyses through TLC-bioautography, LC–MS/MS and NMR allowed the identification of five macrocyclic ionophore antibiotics, with previously reported antibacterial, antitumor and antiviral properties.	Province, Costa Rica	
**(118, 119)**	*Streptomyces davaonensis* YH01	termite *Odontotermes formosanus* (queen, cuticle)	G1 (100 mg cycloheximide)	Roseoflavin and its analog were isolated. The roseoflavin showed strong inhibition activities against gram-positive bacteria.	campus of Zhejiang Normal University (N 29.004825, E 119.498567), Jinhua, Zhejiang, China	[14]
**(23)**	*Pseudonocardia* spp.	ants Attini (*Trachymyrmex*, *Apterostigma*, *Acromyrmex*)	no data	A large systematic study of Brazilian nests led to the discovery of the widespread production of a potent but overlooked antifungal agent, which we named attinimicin, by nearly two-thirds of all *Pseudonocardia* strains from multiple sites in Brazil. Attinimicin shows iron-dependent antifungal activity against specific environmental fungal parasites but no activity against the fungal cultivar. Attinimicin showed potent *in vivo* activity in a mouse *Candida albicans* infection model comparable to clinically used azole-containing antifungals. *In situ* detection of attinimicin in both ant nests and on worker ants supports an ecological role for attinimicin in protecting the fungal cultivar from pathogens. The geographic spread of the attinimicin biosynthetic gene cluster in Brazilian *Pseudonocardia* spp. marks attinimicin as the first specialized metabolite from ant-associated bacteria with broad geographic distribution.	Anavilhanas National Park (Amazonas) and Itatiaia National Park (Rio de Janeiro), Brazil	[15]
**(8)**	*Pseudonocardia* sp.	ants *Trachymyrmex* sp.	no data	Dentigerumycin F was active against	Ducke Reserve,	[16]

Back to contents

	ICBG1122			*Escovopsis* sp. and *Candida albicans* K1	Amazon Region, Brazil	
**(104, 105)**	*Streptomyces* sp. SFA33	ant *Formica yessensis*	mK	Formicins A−C were discovered. Formicin A inhibited the growth of human triple-negative breast cancer cells.	Cheongnyang Mountain, Gwangju-si, Gyeonggi-do, Republic of Korea	[17]
no compounds (only putative)	*Streptomyces* spp.	beetle *Cerambyx welensii* (larvae, gut)	ISP2 and R2YE (nalidixic acid 25 mg, cycloheximide 50 mg)	24 actinomycetes strains isolated from the intestinal tract and feces from larvae of the xylophagous coleopteran. 14 of the isolates produce antimicrobial molecules against the gram-positive bacteria *Micrococcus luteus*.	La Serrana (N 39.023,W 6.638722), Mérida, Spain	[18]
**(19)**	*Streptomyces albidoflavus* A10	ant *Camponotus vagus*	AI (nystatin 250 mg, nalidixic acid 10 mg)	Strain A10 was found to exhibit pronounced antifungal properties, including activity against entomopathogenic fungi, especially against *Conidiobolus coronatus* and *Beauveria bassiana*. The antifungal substances were isolated and identified as antimycin-type compounds.	Kasimovsky District (N 55.01138, E 41.73078), Ryazan region, Russia	[19]
**(20-22)**	*Streptomyces* sp. GG23	beetle *Tenebrio molitor* (gut)	SCN	Pentaminomycins C–E were isolated from the culture. Pentaminomycins C and D showed significant autophagy-inducing activities and were cytoprotective against oxidative stress in vitro.	Seoul Grand Park, Gwacheon-si, Gyeonggi Province, Republic of Korea	[20]
**(18)**	*Streptomyces* sp. GG23 (previously described)	(previously described)	(previously described)	Lydiamycin A displayed weak antituberculosis activity in vitro	(previously described)	[21]
**(17)**	*Pseudonocardia* spp.	ant *Trachymyrmex septentrionalis* (laterocervical plates)	Ch (nystatin 30 mg)	Among nine *Pseudonocardia* strain isolates six were found to produce thiopeptide antibiotic GE37468 (formerly known as an inhibitor of Gram-positive bacteria, e.g. *S.aureus* and *E.faecalis*) thus antagonizing their competitors, other strains of ant-associated *Pseudonocardia* (agar diffusion assay). The compound showed no inhibitory activity on strains *Candida albicans* SC5314 and *Trichoderma harzianum* T22, and neither on *Escovopsis* sp.	Centereach, New York, USA	[22]
**(107, 108)**	*Streptomyces* sp. BA01	ant *Formica yessensis* (gut)	P (cycloheximide 100 mg)	Formicolides A and B showed antioxidative (quinone reductase assay) and	Namhansanseong, Gyenggi-do, Republic	[23]

Back to contents

				antiangiogenic (inhibition of capillary tube formation in human umbilical vein endothelial cells) activity, along with no anti-inflammatory activity (inducible nitric oxide synthase assay).	of Korea	
**(15, 16)**	*Streptomyces* sp. ICBG1318	bee *Melipona scutellaris* (nurse bee)	Ch (cycloheximide 50 mg, nystatin 32 mg)	Meliponamycins A,B showed strong activity against the entomopathogen *Paenibacillus larvae* and human pathogens *Staphylococcus aureus* and *Leishmania infantum*.	Ribeirão Preto (S 21.150000, W 47.850000), São Paulo, Brazil	[24]
**(98-101)**	*Streptomyces* sp. BYF63	termite *Odontotermes formosanus*	M3 (nystatin 75 mg, potassium dichromate 50 mg, nalidixic acid 25 mg)	Termstrins A, B and D exhibited antibacterial effect against *S. aureus*. Termstrin A exhibited moderate cytotoxic activity against the tumor cell lines A375 and MGC-803.	Jiangyin, Jiangsu, China	[25]
**(89-91)**	*Streptomyces* sp. 1H-XA2	ant *Camponotus japonicus* (cuticle)	XA	Furamycins I and II have been reported to show strong antifungal activity. Polyene compound did not show any antifungal or antibacterial activity. Previous study showed that its structural analogs, trichostatin A and BL1521, could inhibit proliferation and induce apoptosis in neuroblastoma cells.	campus of Northeast Agriculture University (N 45.73333, E 126.71667), Harbin, Heilongjiang, China	[26]
**(4), (121)**	*Streptomyces globisporus* WA5-2-37	cockroach *Periplaneta americana* (gut)	G1	Actinomycin X2 and collismycin A showed strong inhibition of MRSA	Guangdong Provincial Center for Disease Control and Prevention, China	[27]
**(81-83), (150)**	*Streptomyces griseus* XylebKG-1	beetle *Xyleborus saxesenii* (female)	Ch (cycloheximide 50 mg, nystatin 30 mg)	In this study we consistently isolated a single *Streptomyces* morphotype and phylotype from both *X. saxesenii* and *X. affinis* that inhibited the growth of the parasitic fungus *Nectria* sp., but not the mutualistic *Raffaelea sulphurea*, likely via the production of cycloheximide. Its ubiquity suggests that XylebKG-1 may be a defensive mutualist of these ambrosia beetles that inhibits the growth of all but a few fungi, including its mutualistic fungal food source. Dihydromaltophilin similarly	Southern Research Station in Pineville (N 31.333, W 92.400), Louisiana, USA	[28]

				inhibited both *Nectria* sp. and *R. sulphurea*. Naramycin B and Actiphenol were non-inhibitory under all conditions tested.		
**(92-96), (148, 149)**	*Streptomyces* sp. N1508.10	Undefined mud dauber wasp (cuticle)	G1 (6 mg potassium dichromate, 20 mg nalidixic acid)	Hexokinase II inhibitory activities of the compounds were evaluated for the first time. As a result, phenylacetic acid, 4-(2-aminoethyl)phenyl acetate, germicidin B, isogermicidin A, and germicidin C displayed significant inhibitory activity against hexokinase II.	Weihai, Shandong Province, China	[29]
**(14)**	*Saccharopolyspora* sp. KY21	ant *Tetraponera penzigi* (cuticle)	no data	Kyamicin has a weak activity against *B. subtilis* EC1524 (MIC 128 ug/ml)	Ngong Hills (S 1.4491,E 36.6393), Kenya	[30]
**(120)**	*Streptomyces* sp. Pe6	ant *Camponotus vagus*	G1 (nalidixic acid 10 mg, nystatin 250 mg)	Nybomycin has cytotoxic activity against human cell lines.	Bryansk Forest Nature State Biosphere Reserve (N 52.440009, E 34.126288), Bryansk region, Russia	[31]
**S_8** (sulphur)	*Streptomyces chartreusis* ICBG323	ant *Mycocepurus goeldii* (exoskeleton of winged male)	Ch (cycloheximide 50 mg, nystatin 40 mg)	Sulphur has antifungal activity against *Escovopsis* sp.	Ribeirão Preto (W 47.848889, S 21.163889), São Paulo state, Brazil	[32]
**(151-165)**	*Streptomyces sp.* ICBG1323 *Micromonospora sp.* ICBG1321	bee *Mellipona scutellaris*	Ch (cycloheximide 50 mg, nystatin 32 mg)	Lobophorins were active against the specialized pathogen of honey bees *Paenibacillus larvae*	Ribeirão Preto (S 21.150000, W 47.850000), São Paulo, Brazil	[33]
**(80), (86), (137, 138)**	*Streptomyces* sp. ICBG292 *Streptomyces* sp. ICBG233 *Streptomyces puniceus* ICBG378 (=AB10, previously described)	ants *Cyphomyrmex* sp. (workers, exoskeleton); *Atta sexdens* (workers, exoskeleton); *Atta rugosus rugosus* (previously described)	Ch (nystatin 40 mg, cycloheximide 50 mg)	Nigericin presented high activity against intracellular amastigotes of *L. donovani* (IC_{50} 0.129 ± 0.008 μM). *Streptomyces puniceus* ICBG378, isolated from workers of *Acromyrmex rugosus rugosus*, produced dinactin with potent anti-*L. donovani* activity against intracellular amastigotes (IC_{50} 0.018 ± 0.003 μM). All compounds were also active against some *Escovopsis* strains. Piericidins were also detected in ants' extracts by mass spectrometry, suggesting they are produced in the natural environment as defensive compounds	campus of the University of São Paulo – Ribeirão Preto (S 21.167889,W 47.847083), Brazil	[34]

Back to contents

				involved in the symbiotic interaction.		
**(52, 53)**	*Streptomyces* sp. SD53	silkworm *Bombyx mori* (larvae, gut)	A1+C (cycloheximide 100 mg)	Bombyxamycin A showed significant antibacterial and antiproliferative effects.	Boeun-eup, Boeun-gun, Chungcheongbuk-do, Republic of Korea	[35]
**(49)**	*Actinomadura* sp. RB99 (previously described)	(previously described)	(previously described)	Fridamycin A induced glucose uptake in 3T3-L1 cells by activating the AMP-activated protein kinase (AMPK) signaling pathway but did not affect adipocyte differentiation	(previously described)	[36]
**(77)**	*Streptomyces* sp.ISID311	ant *Cyphomyrmex* sp.	HV or Ch (no additional data) (nystatin 20 mg, cycloheximide 10 mg)	Cyphomycin shows potent in vitro activity against both the ecologically relevant fungus-growing ant pathogen *Escovopsis* sp. and the resistant human pathogens *Aspergillus fumigatus* 11628 (triazole resistance), *Candida glabrata* 4720 (echinocandin resistance), and *C. auris* B11211 (echinocandin, triazole, and amphotericin B resistance)	no data	[37]
no compounds	*Streptomyces* spp.	bee *Tetragonisca angustula*	ISP3 and Ch agar (nalidixic acid25 mg, cycloheximide or nystatin 50 mg)	Isolates showed antibacterial and antifungal activity.	La Selva Biological Station, Sarapiquí, Costa Rica	[38]
no compounds	*Streptomyces* spp. and *Microbacterium* sp.	ant *Acromyrmex coronatus*	SM3, TS, ISP2, ISP4, Ch (cycloheximide 50 mg, nalidixic acid 10 mg, novobiocin 10 mg, nystatin 50 mg)	Isolates showed insecticidal activity (against *Spodoptera frugiperda* larvae)	Escola Superior de Agricultura "Luiz de Queiroz" in Piracicaba, São Paulo, Brazil	[39]
**(71-73)**	*Streptomyces* sp. N1510.2	wasp *Sceliphron madraspatanum* (larvae)	G1 (potassium dichromate 100 mg, nalidixic acid 20 mg)	Strepantibins A−C inhibited hexokinase II (HK2) activity and displayed antiproliferative activity against hepatoma carcinoma cells HepG-2, SMMC-7721 and plc-prf-5.	Dezhou, Shandong, China	[40]
**(139-140)**	*Streptomyces* sp. STA1	ant *Camponotus kiusiuensis* (gut)	P (cycloheximide 100 mg)	Camporidine A displayed antimetastatic activity by suppression of cell invasion against the metastatic breast cancer cell line MDA-MB-231 and showed an anti-inflammatory effect by suppressing nitric oxide production induced by	Gwanak Mountain, Seoul, Republic of Korea,	[41]

Back to contents

				lipopolysaccharide.		
**(111-117)**	*Streptomyces* sp. NA04227 (previously described)	(previously described)	(previously described)	Compounds exhibited moderate AchE inhibitory activities, and Phenazine SC showed antimicrobial activities against *M. luteus*.	(previously described)	[42]
**(10-12)**	*Actinomadura* sp. RB99	termite *Macrotermes natalensis* (worker, body surface)	Ch	Nataleamide C exhibited significant inhibitory effects on 3-isobutyl-1-methylxanthine (IBMX)-induced melanin production	Pretoria (S 25.729417, E 28.235806), South Africa	[43]
**(68-70)**	*Streptomyces capitiformicae* sp. 1H-SSA4	ant *Camponotus japonicus* (head)	SSA (cycloheximide 50 mg, nalidixic acid 20 mg)	Angucycline antibiotics (8-*O*-methyltetrangulol, 8-*O*-methyltetrangomycin, fugianmycin B) exhibited activities against bacteria and cancer cells	Northeast Agricultural University, Harbin, Heilongjiang, China	[44]
**(146)**	*Streptomyces* sp. RB1 (previously described)	(previously described)	(previously described)	1-*O*-(2-aminobenzoyl)-alpha-L-rhamnopyranoside ameliorated cisplatin-induced cytotoxicity to 80% of the control value at 25 uM by blocking the MAPK signaling cascade.	(previously described)	[45]
**(13)**	*Streptomyces* sp. HKHCa2	grasshopper *Oxya chinensis*	no data	Streptoxamine showed weak antibacterial activity against the gram-positive bacteria, *Staphylococcus aureus* and *Mycobacterium smegmatis*.	Haikou, China	[46]
no compounds	*Streptomyces lasii* 5H-CA11	ant *Lasius flavus* (head)	Ch (cycloheximide 50 mg, nalidixic acid 20 mg)	Strain exhibited antifungal activity	Northeast Agriculture University, Harbin, Heilongjiang, China	[47]
**(131-136)**	*Actinomadura rubteroloni* RB29 (= *Actinomadura* sp.5-2) (previously described)	(previously described)	(previously described)	Rubterolones and related compounds have anti-inflammatory activity	(previously described)	[48]
**(9)**	*Actinomadura rubteroloni* RB29 (= *Actinomadura* sp.5-2) (previously described)	(previously described)	(previously described)	cyclo(*N*Me-L-3,5-dichlorotyrosin-Dhb) has antibacterial activity	(previously described)	[49]
**(125-130)**	*Actinomadura* sp. 5-2 (=*Actinomadura rubteroloni* RB29)	termite *Macrotermes natalensis* (gut)	Ch or MC (cycloheximide 50 mg) (no additional data)	Strain 5-2 (RB29) displayed weak antimicrobial activity against *B. subtilis* and moderate to good antifungal activity in co-cultivation assay.	Pretoria (S 25.743278, E 28.263333), South Africa	[50]
**(122-124)**	*Streptomyces* sp.	earwig *Forficula*	ISP4 (with	Compounds showed moderate activity	Qixia Mountain in the	[51]

	NA04227	*auricularia*	cycloheximide)	against Gram-positive bacteria	suburb of Nanjing, Jiangsu, China	
**(67)**	*Streptomyces amphotericinicus* 1H-SSA8	ant *Camponotus japonicus* (head)	SSA (cycloheximide 50 mg, nalidixic acid 20 mg)	Strain 1H-SSA8T showed broad-spectrum antifungal activity. The strain was found to produce amphotericin.	Northeast Agriculture University, Harbin, Heilongjiang, China	[52]
**(76)**	*Streptomyces lasiicapitis* 3H-HV17(2)	ant *Lasius fuliginosus* (head)	HV (cycloheximide 50 mg, nalidixic acid 20 mg)	Kanchanamycin is a potential antifungal agent.	Northeast Agriculture University, Harbin, Heilongjiang, China	[53]
**(50, 51)**	*Streptomyces puniceus* AB10 (=*Streptomyces puniceus* ICBG378)	ant *Atta rugosus rugosus* (worker)	Ch (nystatin 40 mg, cycloheximide 50 mg)	no data	campus of the University of São Paulo – Ribeirão Preto (S 21.167889,W 47.847083), Brazil	[54]
**(54-66)**	*Streptomyces formicae* KY5	ant *Tetraponera penzigi*	no data	Novel pentacyclic polyketides are active against MRSA and VRE. The formicamycins are more potent than the previously reported and structurally related fasamycins.	Kenya (no additional data)	[55]
**(39)**	*Streptomyces* sp. 4231	termite *Coptotermes formosanus* (fecal and wood material)	TFA (cycloheximide 100 mg)	The release of bafilomycin C1 by *Streptomyces* sp. 4231 induces the production of several defensive metabolites by *Trichoderma harzianum* WC13.	Broward County, Florida, USA	[56]
**(2, 3)**	*Microbacterium* sp. UTG9	beetle *Nicrophorus concolor* (gut)	SC (cycloheximide 100 mg)	Nicrophorusamide A showed antibacterial activity against several gram-positive bacteria.	Maebong Mountain, Seoul, Republic of Korea	[57]
**(40, 41), (47, 48)**	*Amycolatopsis* sp. M39	termite *Macrotermes natalensis* (worker)	MC	Macrotermycins A and C had antibacterial activity against human-pathogenic *Staphylococcus aureus* and, of greater ecological relevance, they also had selective antifungal activity against a fungal parasite of the termite fungal garden.	South Africa (no additional data)	[58]
**(5-7)**	*Streptomyces* sp. M41	termite *Macrotermes natalensis*	no data	Dentigerumycins B-D were screened against a panel of gram-positive and gram-negative bacteria, yeast, and fungi, but no activity was detected.	no data	[59]
**(144-145)**	*Amycolatopsis* sp. HCa4 (previously described)	(previously described)	(previously described)	Amycolamycin A suppressed the proliferation of M231 cells by inducing apoptosis through activation of caspase-3.	(previously described)	[60]
**(42-46)**	*Amycolatopsis* sp.	locust *Locusta*	G1	Compounds have activity against gram-	Zijing Mountain,	[61]

	HCa4	*migratoria*		positive bacteria	Nanjing, China	
no compounds	*Streptomyces* spp.	beetle *Orthotomicus erosus*	Ch (cycloheximide 5 mg, nystatin 10 kU)	11 *Streptomyces* spp. isolates with antifungal activity were collected from adult beetles that infest *Pinus* spp.	Lothair plantation, Mpumalanga Province, South Africa	[62]
no compounds	*Streptomyces* spp.	termites *Reticulitermes flavipes*, *Reticulitermes tibialis* (guts)	Ch (nystatin 43 mg, cyclohexamide 67 mg)	Actinobacteria expressed a diversity of antimicrobial characteristics against mold fungi and *Serratia marcescens* showed a broad range of antimicrobial activity in vitro (34 from 38 were active against 1 test-culture at least).	southern Wisconsin (Janesville, Muscoda, Hazel Green), USA	[63]
**(1)**	*Streptomyces sp.* IB2014/010-1	caddisfly *Trichoptera* sp. (larvae)	MS or Cz (cycloheximide 50 mg, phosphomycin 100 mg) (no additional data)	Variapeptin is a hexadepsipeptide antibiotic with antibacterial activity of azinothricin family.	South Baikal near Listvyanka village (N 51.867936, E 104.829715), Irkutsk region, Russia	[64]
no compounds	*Pseudonocardia* spp.	ant *Acromyrmex echinatior* (workers, laterocervical plates)	LB	Strains show antibacterial antagonistic activity.	Soberania National Park, Gamboa, Panama	[65]
**(34, 35)**	*Streptomyces* sp. 1H-GS5	ant *Camponotus japonicus* (head)	no data	Spectinabiblin derivative 1 exhibited good cytotoxic activities.	Northeast Agricultural University, Harbin, Heilongjiang, China	[66]
**(36-38)**	*Streptomyces* sp. 1H-GS5 (previously described)	(previously described)	(previously described)	A new spectinabilin derivative showed cytotoxicity against human tumor cell lines.	(previously described)	[67]
**(141-143), (147)**	*Streptomyces* sp. RB1	termite *Macrotermes natalensis* (worker, cuticle)	no data	*Streptomyces* sp. RB1 showed significant activity against *Staphylococcus aureus* and weak activity against human-pathogenic *Candida albicans*. Three new isoflavonoid glycosides, termisoflavones A−C, and eight isoflavonoids were isolated. None of the isolated compounds displayed antifungal or antimicrobial activities.	Pretoria, South Africa	[68]
**(109, 110)**	*Streptomyces drozdowiczii* SNU607	beetle *Copris tripartitus* (gut)	P (cycloheximide 200 mg)	Coprisidin A was found to inhibit the action of Na+/K+-ATPase, and coprisidin B showed activity for the induction of NAD(P)H:quinone oxidoreductase 1 (NQO1).	Jeju Island (N 33.385359, E 126.732773), Republic of Korea	[69]
**(33)**	*Pseudonocardia* spp.	ants *Apterostigma* sp.	no data	Novel antifungal polyene	La Selva Biological Station, Costa Rica	[70]

no compounds	*Streptomyces formicae* 1H-GS9	ant *Camponotus japonicus* (head)	G1 (cycloheximide 50 mg, nalidixic acid 20 mg)	Strain 1H-GS9T was found to have antifungal activity.	Northeast Agricultural University, Harbin, Heilongjiang, China	[71]

TABLE S2. MEDIA USED FOR ACTINOBACTERIA ISOLATION

code	Full name, synonyms	Ingredients (per 1 L)	Reference
A1	DSMZ 1054	soluble starch 10 g, yeast extract 4 g, peptone 2 g	[72] p.11
A1+C	-	soluble starch 10 g, yeast extract 4 g, peptone 2 g, $CaCO_3$ 1 g	[35]
AI	Actinomycete isolation medium, Streptomycete isolation medium	glycerol 5 g, sodium propionate 4 g, sodium caseinate 2 g, K_2HPO_4 0.5g, asparagine 0.1 g, $MgSO_4 \times 7H_2O$ 0.1g, $FeSO_4 \times 7H_2O$ 1 mg	[72] p.57
Ch	Chitin medium	chitin 4 g; K_2HPO_4 0.7 g; KH_2PO_4 0.3 g; $MgSO_4 \times 5H_2O$ 0.5 g; $FeSO_4 \times 7H_20$ 10 mg; $ZnSO_4$ 1 mg; $MnCl_2$ 1 mg	[73]
Cz	Czapek medium	sucrose 30 g, $NaNO_3$ 3 g, K_2HPO_4 1 g, KCl 0.5 g, $MgSO_4 \times 7H_2O$ 0.5 g, $FeSO_4 \times 7H_2O$ 0.01 g	[72] p.479
G1	Gause medium #1, Gause synthetic medium, DSMZ 1048	soluble starch 20 g, KNO_3 1 g, K_2HPO_4 0.5g, $MgSO_4$ 0.5 g, NaCl 0.5 g, $FeSO_4$ 0.01g	[72] p.719
HV	Humic medium, Humic Acid-Vitamin medium	humic acid 1 g, Na_2HPO_4 0.5 g, KCI 1.71 g, $MgSO_4 \times 7H_2O$ 0.05 g, $FeSO_4 \times 7H_2O$ 0.01 g, $CaCO_3$ 0.02 g, B-vitamins	[74]
ISP2	International Streptomyces Project medium #2, Yeast extract-malt extract agar	yeast extract 4 g, malt extract 10 g, glucose 4 g	[75]
ISP3	International Streptomyces Project medium #3, Oatmeal medium	oatmeal 20 g, trace elements	[75]
ISP4	International Streptomyces Project medium #4, Starch ammonia medium, Inorganic salts-starch medium	soluble starch 10 g, K_2HPO_4 1 g, $MgSO_4 \times 7H_2O$ 1.0 g, NaCl 1.0 g, $(NH_4)_2SO_4$ 2 g, $CaCO_3$ 2 g, trace elements	[75]
LB	Lysogeny broth, Luria broth, Lennox broth, Luria-Bertani medium	tryptone10 g, yeast extract 5 g, NaCl 10 g	[72] p.911
M3	-	monosodium citrate 0.12 g, citric acid monohydrate 0.12 g, $NaNO_3$ 1.5 g, $K_2HPO_4 \times H_2O$ 0.4 g, $MgSO_4 \times 7H_2O$ 0.1 g, $CaCl_2 \times H_2O$ 0.05 g, EDTA 0.02 g, Na_2CO_3 0.2 g	[76]
MC	Microcrystalline cellulose medium, Microcrystalline medium	microcrystalline cellulose 5 g	[77]
mK	modified K medium	yeast extract 4 g, malt extract 5 g, soytone 5 g, soluble starch 5 g, mannitol 5 g, glucose 2 g	[78]
MS	Mannitol-soy medium	soy flour 20 g; D-mannitol 20 g	[79] p.409
P	Pure agar	no additives	-
PD	Potato-dextrose medium, Potato-glucose medium	infusion from 200 g of unpeeled potatoes, glucose 20 g	[72] p.1412
PY-CMC	ATCC medium 1513, Peptone Yeast Extract Carboxymethyl Cellulose Medium	carboxymethyl cellulose 10 g, peptone 5 g, yeast extract 5 g, NaCl 5 g, KH_2PO_4 1 g, $MgSO_4 \times 7H_2O$ 2 g, Na_2CO_3 10 g	[72] p.1451
R2YE	R2 with yeast extract	sucrose 103 g, K_2SO_4 0.25 g, $MgCl_2 \times 6H_2O$ 10.12 g, glucose 10 g, Difco Casaminoacids 0.1 g, yeast extract 5 g, KH_2PO_4 50 mg, $CaCl_2 \times 2H_2O$, L-proline, TES buffer, trace elements, NaOH	[79] p.408
SCN	Starch-casein-nitrate medium	soluble starch 10 g, casein 0.3 g, KNO_3 2 g, $MgSO_4 \times 7H_2O$ 0.05 g, K_2HPO_4 2 g, NaCl 2 g, $CaCO_3$ 0.02 g, $FeSO_4 \times 7H_2O$ 0.01 g	[72] p.1635-1636

Back to contents

SC	Starch-casein medium	soluble starch 10 g, casein 1 g, K_2HPO_4 0.5 g	[80]
SM3	*Amycolatopsis* selective medium #3	glucose 10 g; peptone 5 g; tryptone 3 g; NaCl 5 g; supplemented with filter sterilised solutions of cycloheximide 50 mg, nalidixic acid 10 mg, novobiocin 10 mg and nystatin 50 mg	[81]
SSA	Sodium succinate-asparagine medium	asparagine 0.2 g; sodium succinate 1 g; $CaCl_2 \times 2H_2O$ 0.2 g; $FeSO_4 \times 7H_2O$ 1 mg; KCl 0.3 g; KH_2PO_4 0.9 g; $K_2HPO_4 \times 3H_2O$ 0.6 g	[52]
TS	Trypticase soy medium, ATCC medium 18, Soybean-casein digest medium	pancreatic digest of casein 15 g, papaic digest of soybean meal 5 g, NaCl 5 g	[72] p.1825
TFA	Termite faecal medium	ground carton material from a *Coptotermes formosanus* laboratory nest 60 g	[82]
XA	Xylan-arginine medium	xylan 2.5g, arginine 1 g, $(NH_4)_2SO_4$ 1 g, $CaCl_2$ 2 g, K_2HPO_4 1 g, $MgSO_4 \times 7H_2O$ 0.2 g, $FeSO_4 \times 7H_2O$ 10 mg	[83]

FIGURE S1. MEDIA USED FOR ACTINOBACTERIA ISOLATION

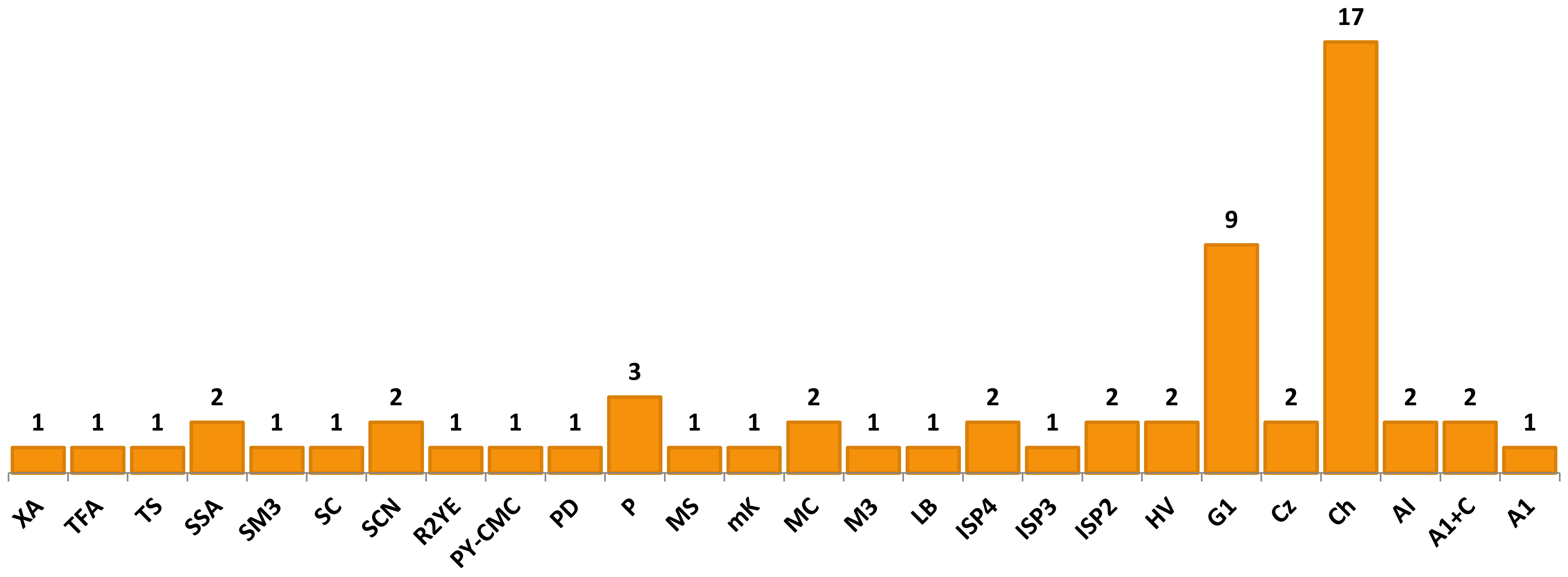

Labels in the histogram are identical to those in Table S2

Back to contents

LIST S1. REFERENCES FOR TABLES S1 AND S2

1. Zakalyukina, Y.V.; Osterman, I.A.; Wolf, J.; Neumann-Schaal, M.; Nouioui, I.; Biryukov, M.V. *Amycolatopsis Camponoti* Sp. Nov., New Tetracenomycin-Producing Actinomycete Isolated from Carpenter Ant *Camponotus Vagus*. *Antonie Leeuwenhoek* **2022**, *115*, 533–544, doi:10.1007/s10482-022-01716-w.
2. An, J.S.; Lim, H.-J.; Lee, J.Y.; Jang, Y.-J.; Nam, S.-J.; Lee, S.K.; Oh, D.-C. Hamuramicin C, a Cytotoxic Bicyclic Macrolide Isolated from a Wasp Gut Bacterium. *J. Nat. Prod.* **2022**, *85*, 936–942, doi:10.1021/acs.jnatprod.1c01075.
3. Wu, Y.; Liu, Y.; Yu, J.; Xu, Y.; Chen, S. Observation of the Antimicrobial Activities of Two Actinomycetes in the Harvester Ant *Messor Orientalis*. *Insects* **2022**, *13*, 691, doi:10.3390/insects13080691.
4. Wu, W.; Chen, B.; Zhang, S.; Guo, Z.; Li, J. Isolation and Identification of Symbiotic Actinomycetes from Termites in Hainan and Their Activity Against Tropical Plants Pathogenic Fungi. *Chin. J. Trop. Crops* **2022**, *43*, 1240–1247, doi:10.3969/j.issn.1000-2561.2022.06.017.
5. Lee, S.R.; Schalk, F.; Schwitalla, J.W.; Guo, H.; Yu, J.S.; Song, M.; Jung, W.H.; de Beer, Z.W.; Beemelmanns, C.; Kim, K.H. GNPS-Guided Discovery of Madurastatin Siderophores from the Termite-Associated *Actinomadura* Sp. RB99**. *Chem. Eur. J.* **2022**, *28*, doi:10.1002/chem.202200612.
6. Promnuan, Y.; Promsai, S.; Pathom-aree, W.; Meelai, S. *Apis Andreniformis* Associated Actinomycetes Show Antimicrobial Activity against Black Rot Pathogen (*Xanthomonas Campestris* Pv. *Campestris*). *PeerJ* **2021**, *9*, e12097, doi:10.7717/peerj.12097.
7. Dhodary, B.; Spiteller, D. Ammonia Production by *Streptomyces* Symbionts of *Acromyrmex* Leaf-Cutting Ants Strongly Inhibits the Fungal Pathogen *Escovopsis*. *Microorganisms* **2021**, *9*, 1622, doi:10.3390/microorganisms9081622.
8. Shin, Y.-H.; Ban, Y.H.; Shin, J.; Park, I.W.; Yoon, S.; Ko, K.; Shin, J.; Nam, S.-J.; Winter, J.M.; Kim, Y.; et al. Azetidine-Bearing Non-Ribosomal Peptides, Bonnevillamides D and E, Isolated from a Carrion Beetle-Associated Actinomycete. *J. Org. Chem.* **2021**, *86*, 11149–11159, doi:10.1021/acs.joc.1c00360.
9. Shin, Y.-H.; Ban, Y.H.; Kim, T.H.; Bae, E.S.; Shin, J.; Lee, S.K.; Jang, J.; Yoon, Y.J.; Oh, D.-C. Structures and Biosynthetic Pathway of Coprisamides C and D, 2-Alkenylcinnamic Acid-Containing Peptides from the Gut Bacterium of the Carrion Beetle *Silpha Perforata*. *J. Nat. Prod.* **2021**, *84*, 239–246, doi:10.1021/acs.jnatprod.0c00864.
10. Li, J.; Sang, M.; Jiang, Y.; Wei, J.; Shen, Y.; Huang, Q.; Li, Y.; Ni, J. Polyene-Producing *Streptomyces* Spp. From the Fungus-Growing Termite *Macrotermes Barneyi* Exhibit High Inhibitory Activity Against the Antagonistic Fungus *Xylaria*. *Front. Microbiol.* **2021**, *12*, 649962, doi:10.3389/fmicb.2021.649962.
11. Xu, Y.; Xie, J.; Wu, W.C.; Chen, B.T.; Zhang, S.Q.; Wang, R.; Huang, J.; Guo, Z.K. Discovery of an Unprecedented Benz[a]Anthraquinone-Type Heterodimer from a Rare Actinomycete *Amycolatopsis* Sp. HCa1. *Fitoterapia* **2021**, *155*, 105039, doi:10.1016/j.fitote.2021.105039.
12. Ortega, H.E.; Lourenzon, V.B.; Chevrette, M.G.; Ferreira, L.L.G.; Alvarenga, R.F.R.; Melo, W.G.P.; Venâncio, T.; Currie, C.R.; Andricopulo, A.D.; Bugni, T.S.; et al. Antileishmanial Macrolides from Ant-Associated *Streptomyces* Sp. ISID311. *Bioorg. Med. Chem.* **2021**, *32*, 116016, doi:10.1016/j.bmc.2021.116016.
13. Matarrita-Carranza, B.; Murillo-Cruz, C.; Avendaño, R.; Ríos, M.I.; Chavarría, M.; Gómez-Calvo, M.L.; Tamayo-Castillo, G.; Araya, J.J.; Pinto-Tomás, A.A. *Streptomyces* Sp. M54: An Actinobacteria Associated with a Neotropical Social Wasp with High Potential for Antibiotic Production. *Antonie Leeuwenhoek* **2021**, *114*, 379–398, doi:10.1007/s10482-021-01520-y.
14. Zhou, L.-F.; Wu, J.; Li, S.; Li, Q.; Jin, L.-P.; Yin, C.-P.; Zhang, Y.-L. Antibacterial Potential of Termite-Associated *Streptomyces* Spp. *ACS Omega* **2021**, *6*, 4329–4334, doi:10.1021/acsomega.0c05580.

15. Fukuda, T.T.H.; Helfrich, E.J.N.; Mevers, E.; Melo, W.G.P.; Van Arnam, E.B.; Andes, D.R.; Currie, C.R.; Pupo, M.T.; Clardy, J. Specialized Metabolites Reveal Evolutionary History and Geographic Dispersion of a Multilateral Symbiosis. *ACS Cent. Sci.* **2021**, *7*, 292–299, doi:10.1021/acscentsci.0c00978.
16. Bae, M.; Mevers, E.; Pishchany, G.; Whaley, S.G.; Rock, C.O.; Andes, D.R.; Currie, C.R.; Pupo, M.T.; Clardy, J. Chemical Exchanges between Multilateral Symbionts. *Org. Lett.* **2021**, *23*, 1648–1652, doi:10.1021/acs.orglett.1c00068.
17. Du, Y.E.; Byun, W.S.; Lee, S.B.; Hwang, S.; Shin, Y.-H.; Shin, B.; Jang, Y.-J.; Hong, S.; Shin, J.; Lee, S.K.; et al. Formicins, *N*-Acetylcysteamine-Bearing Indenone Thioesters from a Wood Ant-Associated Bacterium. *Org. Lett.* **2020**, *22*, 5337–5341, doi:10.1021/acs.orglett.0c01584.
18. Santamaría, R.I.; Martínez-Carrasco, A.; Sánchez de la Nieta, R.; Torres-Vila, L.M.; Bonal, R.; Martín, J.; Tormo, R.; Reyes, F.; Genilloud, O.; Díaz, M. Characterization of Actinomycetes Strains Isolated from the Intestinal Tract and Feces of the Larvae of the Longhorn Beetle *Cerambyx Welensii*. *Microorganisms* **2020**, *8*, 2013, doi:10.3390/microorganisms8122013.
19. Baranova, A.A.; Chistov, A.A.; Tyurin, A.P.; Prokhorenko, I.A.; Korshun, V.A.; Biryukov, M.V.; Alferova, V.A.; Zakalyukina, Y.V. Chemical Ecology of *Streptomyces Albidoflavus* Strain A10 Associated with Carpenter Ant Camponotus Vagus. *Microorganisms* **2020**, *8*, 1948, doi:10.3390/microorganisms8121948.
20. Hwang, S.; Le, L.T.H.L.; Jo, S.-I.; Shin, J.; Lee, M.J.; Oh, D.-C. Pentaminomycins C–E: Cyclic Pentapeptides as Autophagy Inducers from a Mealworm Beetle Gut Bacterium. *Microorganisms* **2020**, *8*, 1390, doi:10.3390/microorganisms8091390.
21. Hwang, S.; Shin, D.; Kim, T.H.; An, J.S.; Jo, S.-I.; Jang, J.; Hong, S.; Shin, J.; Oh, D.-C. Structural Revision of Lydiamycin A by Reinvestigation of the Stereochemistry. *Org. Lett.* **2020**, *22*, 3855–3859, doi:10.1021/acs.orglett.0c01110.
22. Chang, P.T.; Rao, K.; Longo, L.O.; Lawton, E.S.; Scherer, G.; Van Arnam, E.B. Thiopeptide Defense by an Ant's Bacterial Symbiont. *J. Nat. Prod.* **2020**, *83*, 725–729, doi:10.1021/acs.jnatprod.9b00897.
23. An, J.S.; Lee, J.Y.; Kim, E.; Ahn, H.; Jang, Y.-J.; Shin, B.; Hwang, S.; Shin, J.; Yoon, Y.J.; Lee, S.K.; et al. Formicolides A and B, Antioxidative and Antiangiogenic 20-Membered Macrolides from a Wood Ant Gut Bacterium. *J. Nat. Prod.* **2020**, *83*, 2776–2784, doi:10.1021/acs.jnatprod.0c00772.
24. Menegatti, C.; Lourenzon, V.B.; Rodríguez-Hernández, D.; da Paixão Melo, W.G.; Ferreira, L.L.G.; Andricopulo, A.D.; do Nascimento, F.S.; Pupo, M.T. Meliponamycins: Antimicrobials from Stingless Bee-Associated *Streptomyces* Sp. *J. Nat. Prod.* **2020**, *83*, 610–616, doi:10.1021/acs.jnatprod.9b01011.
25. Zhang, L.; Song, T.; Wu, J.; Zhang, S.; Yin, C.; Huang, F.; Hang, Y.; Abbas, N.; Liu, X.; Zhang, Y. Antibacterial and Cytotoxic Metabolites of Termite-Associated *Streptomyces* Sp. BYF63. *J. Antibiot.* **2020**, *73*, 766–771, doi:10.1038/s41429-020-0334-1.
26. Wang, Z.; Yu, Z.; Zhao, J.; Zhuang, X.; Cao, P.; Guo, X.; Liu, C.; Xiang, W. Community Composition, Antifungal Activity and Chemical Analyses of Ant-Derived Actinobacteria. *Front. Microbiol.* **2020**, *11*, 201, doi:10.3389/fmicb.2020.00201.
27. Chen, Z.; Ou, P.; Liu, L.; Jin, X. Anti-MRSA Activity of Actinomycin X2 and Collismycin A Produced by *Streptomyces Globisporus* WA5-2-37 From the Intestinal Tract of American Cockroach (*Periplaneta Americana*). *Front. Microbiol.* **2020**, *11*, 555, doi:10.3389/fmicb.2020.00555.
28. Grubbs, K.J.; Surup, F.; Biedermann, P.H.W.; McDonald, B.R.; Klassen, J.L.; Carlson, C.M.; Clardy, J.; Currie, C.R. Cycloheximide-Producing *Streptomyces* Associated With *Xyleborinus Saxesenii* and *Xyleborus Affinis* Fungus-Farming Ambrosia Beetles. *Front. Microbiol.* **2020**, *11*, 562140, doi:10.3389/fmicb.2020.562140.
29. Zhang, X.; Peng, A.; Xie, W.; Wang, M.; Zheng, D.; Feng, M. Hexokinase II Inhibitory Effect of Secondary Metabolites Derived from a *Streptomyces* Sp. Associated with Mud Dauber Wasp. *Chem. Biodivers.* **2020**, *17*, e2000140, doi:10.1002/cbdv.202000140.
30. Vikeli, E.; Widdick, D.A.; Batey, S.F.D.; Heine, D.; Holmes, N.A.; Bibb, M.J.; Martins, D.J.; Pierce, N.E.; Hutchings, M.I.; Wilkinson, B. *In Situ* Activation and Heterologous Production of a Cryptic Lantibiotic from an African Plant Ant-Derived *Saccharopolyspora* Species. *Appl. Environ. Microbiol.* **2020**, *86*, e01876-19, doi:10.1128/AEM.01876-19.

Back to contents

31. Zakalyukina, Y.V.; Birykov, M.V.; Lukianov, D.A.; Shiriaev, D.I.; Komarova, E.S.; Skvortsov, D.A.; Kostyukevich, Y.; Tashlitsky, V.N.; Polshakov, V.I.; Nikolaev, E.; et al. Nybomycin-Producing *Streptomyces* Isolated from Carpenter Ant *Camponotus Vagus*. *Biochimie* **2019**, *160*, 93–99, doi:10.1016/j.biochi.2019.02.010.
32. Ortega, H.; Batista, J.; Melo, W.; de Paula, G.; Pupo, M. Structure and Absolute Configuration of Secondary Metabolites from Two Strains of *Streptomyces Chartreusis* Associated with Attine Ants. *J. Braz. Chem. Soc.* **2019**, doi:10.21577/0103-5053.20190194.
33. Rodríguez-Hernández, D.; Melo, W.G.P.; Menegatti, C.; Lourenzon, V.B.; do Nascimento, F.S.; Pupo, M.T. Actinobacteria Associated with Stingless Bees Biosynthesize Bioactive Polyketides against Bacterial Pathogens. *New J. Chem.* **2019**, *43*, 10109–10117, doi:10.1039/C9NJ01619H.
34. Ortega, H.E.; Ferreira, L.L.G.; Melo, W.G.P.; Oliveira, A.L.L.; Ramos Alvarenga, R.F.; Lopes, N.P.; Bugni, T.S.; Andricopulo, A.D.; Pupo, M.T. Antifungal Compounds from *Streptomyces* Associated with Attine Ants Also Inhibit *Leishmania Donovani*. *PLoS Negl. Trop. Dis.* **2019**, *13*, e0007643, doi:10.1371/journal.pntd.0007643.
35. Shin, Y.-H.; Beom, J.Y.; Chung, B.; Shin, Y.; Byun, W.S.; Moon, K.; Bae, M.; Lee, S.K.; Oh, K.-B.; Shin, J.; et al. Bombyxamycins A and B, Cytotoxic Macrocyclic Lactams from an Intestinal Bacterium of the Silkworm *Bombyx Mori*. *Org. Lett.* **2019**, *21*, 1804–1808, doi:10.1021/acs.orglett.9b00384.
36. Yoon, S.-Y.; Lee, S.R.; Hwang, J.Y.; Benndorf, R.; Beemelmanns, C.; Chung, S.J.; Kim, K.H. Fridamycin A, a Microbial Natural Product, Stimulates Glucose Uptake without Inducing Adipogenesis. *Nutrients* **2019**, *11*, 765, doi:10.3390/nu11040765.
37. Chevrette, M.G.; Carlson, C.M.; Ortega, H.E.; Thomas, C.; Ananiev, G.E.; Barns, K.J.; Book, A.J.; Cagnazzo, J.; Carlos, C.; Flanigan, W.; et al. The Antimicrobial Potential of *Streptomyces* from Insect Microbiomes. *Nat. Commun.* **2019**, *10*, 516, doi:10.1038/s41467-019-08438-0.
38. Cambronero-Heinrichs, J.C.; Matarrita-Carranza, B.; Murillo-Cruz, C.; Araya-Valverde, E.; Chavarría, M.; Pinto-Tomás, A.A. Phylogenetic Analyses of Antibiotic-Producing *Streptomyces* Sp. Isolates Obtained from the Stingless-Bee *Tetragonisca Angustula* (Apidae: Meliponini). *Microbiology* **2019**, *165*, 292–301, doi:10.1099/mic.0.000754.
39. Martinez, A.F.C.; de Almeida, L.G.; Moraes, L.A.B.; Cônsoli, F.L. Microbial Diversity and Chemical Multiplicity of Culturable, Taxonomically Similar Bacterial Symbionts of the Leaf-Cutting Ant *Acromyrmex Coronatus*. *Microb. Ecol.* **2019**, *77*, 1067–1081, doi:10.1007/s00248-019-01341-7.
40. Song, Y.-J.; Zheng, H.-B.; Peng, A.-H.; Ma, J.-H.; Lu, D.-D.; Li, X.; Zhang, H.-Y.; Xie, W.-D. Strepantibins A–C: Hexokinase II Inhibitors from a Mud Dauber Wasp Associated *Streptomyces* Sp. *J. Nat. Prod.* **2019**, *82*, 1114–1119, doi:10.1021/acs.jnatprod.8b00821.
41. Hong, S.-H.; Ban, Y.H.; Byun, W.S.; Kim, D.; Jang, Y.-J.; An, J.S.; Shin, B.; Lee, S.K.; Shin, J.; Yoon, Y.J.; et al. Camporidines A and B: Antimetastatic and Anti-Inflammatory Polyketide Alkaloids from a Gut Bacterium of *Camponotus Kiusiuensis*. *J. Nat. Prod.* **2019**, *82*, 903–910, doi:10.1021/acs.jnatprod.8b01000.
42. Han, H.; Guo, Z.-K.; Zhang, B.; Zhang, M.; Shi, J.; Li, W.; Jiao, R.-H.; Tan, R.-X.; Ge, H.-M. Bioactive Phenazines from an Earwig-Associated *Streptomyces* Sp. *Chin. J. Nat. Med.* **2019**, *17*, 475–480, doi:10.1016/S1875-5364(19)30055-X.
43. Lee, S.R.; Lee, D.; Yu, J.S.; Benndorf, R.; Lee, S.; Lee, D.-S.; Huh, J.; de Beer, Z.W.; Kim, Y.H.; Beemelmanns, C.; et al. Natalenamides A–C, Cyclic Tripeptides from the Termite-Associated *Actinomadura* Sp. RB99. *Molecules* **2018**, *23*, 3003, doi:10.3390/molecules23113003.
44. Jiang, S.; Piao, C.; Yu, Y.; Cao, P.; Li, C.; Yang, F.; Li, M.; Xiang, W.; Liu, C. *Streptomyces Capitiformicae* Sp. Nov., a Novel Actinomycete Producing Angucyclinone Antibiotics Isolated from the Head of *Camponotus Japonicus* Mayr. *Int. J. Syst. Evol. Microbiol.* **2018**, *68*, 118–124, doi:10.1099/ijsem.0.002468.
45. Lee, D.; Kang, K.; Lee, H.-J.; Kim, K. Chemical Characterization of a Renoprotective Metabolite from Termite-Associated *Streptomyces* Sp. RB1 against Cisplatin-Induced Cytotoxicity. *Int. J. Mol. Sci.* **2018**, *19*, 174, doi:10.3390/ijms19010174.

Back to contents

46. Guo, Z.K.; Wang, R.; Chen, F.X.; Liu, T.M. Streptoxamine, an Unprecedented Benzoisoindole-Deferoxamine Hybrid from the Locust-Derived *Streptomyces* Sp. HKHCa2. *Fitoterapia* **2018**, *127*, 25–28, doi:10.1016/j.fitote.2018.03.008.
47. Liu, C.; Han, C.; Jiang, S.; Zhao, X.; Tian, Y.; Yan, K.; Wang, X.; Xiang, W. *Streptomyces Lasii* Sp. Nov., a Novel Actinomycete with Antifungal Activity Isolated from the Head of an Ant (*Lasius Flavus*). *Curr. Microbiol.* **2018**, *75*, 353–358, doi:10.1007/s00284-017-1388-6.
48. Guo, H.; Benndorf, R.; König, S.; Leichnitz, D.; Weigel, C.; Peschel, G.; Berthel, P.; Kaiser, M.; Steinbeck, C.; Werz, O.; et al. Expanding the Rubterolone Family: Intrinsic Reactivity and Directed Diversification of PKS-Derived Pyrans. *Chem. Eur. J.* **2018**, *24*, 11319–11324, doi:10.1002/chem.201802066.
49. Benndorf, R.; Guo, H.; Sommerwerk, E.; Weigel, C.; Garcia-Altares, M.; Martin, K.; Hu, H.; Küfner, M.; de Beer, Z.; Poulsen, M.; et al. Natural Products from Actinobacteria Associated with Fungus-Growing Termites. *Antibiotics* **2018**, *7*, 83, doi:10.3390/antibiotics7030083.
50. Guo, H.; Benndorf, R.; Leichnitz, D.; Klassen, J.L.; Vollmers, J.; Görls, H.; Steinacker, M.; Weigel, C.; Dahse, H.-M.; Kaster, A.-K.; et al. Isolation, Biosynthesis and Chemical Modifications of Rubterolones A-F: Rare Tropolone Alkaloids from *Actinomadura* Sp. 5-2. *Chem. Eur. J.* **2017**, *23*, 9338–9345, doi:10.1002/chem.201701005.
51. Zhang, M.; Yang, C.L.; Xiao, Y.S.; Zhang, B.; Deng, X.Z.; Yang, L.; Shi, J.; Wang, Y.S.; Li, W.; Jiao, R.H.; et al. Aurachin SS, a New Antibiotic from *Streptomyces* Sp. NA04227. *J. Antibiot.* **2017**, *70*, 853–855, doi:10.1038/ja.2017.50.
52. Cao, T.; Mu, S.; Lu, C.; Zhao, S.; Li, D.; Yan, K.; Xiang, W.; Liu, C. *Streptomyces Amphotericinicus* Sp. Nov., an Amphotericin-Producing Actinomycete Isolated from the Head of an Ant (*Camponotus Japonicus* Mayr). *Int. J. Syst. Evol. Microbiol.* **2017**, *67*, 4967–4973, doi:10.1099/ijsem.0.002382.
53. Ye, L.; Zhao, S.; Li, Y.; Jiang, S.; Zhao, Y.; Li, J.; Yan, K.; Wang, X.; Xiang, W.; Liu, C. *Streptomyces Lasiicapitis* Sp. Nov., an Actinomycete That Produces Kanchanamycin, Isolated from the Head of an Ant (*Lasius Fuliginosus* L.). *Int. J. Syst. Evol. Microbiol.* **2017**, *67*, 1529–1534, doi:10.1099/ijsem.0.001756.
54. Ortega, H.E.; Batista, J.M.; Melo, W.G.P.; Clardy, J.; Pupo, M.T. Absolute Configurations of Griseorhodins A and C. *Tetrahedron Lett.* **2017**, *58*, 4721–4723, doi:10.1016/j.tetlet.2017.11.008.
55. Qin, Z.; Munnoch, J.T.; Devine, R.; Holmes, N.A.; Seipke, R.F.; Wilkinson, K.A.; Wilkinson, B.; Hutchings, M.I. Formicamycins, Antibacterial Polyketides Produced by *Streptomyces Formicae* Isolated from African *Tetraponera* Plant-Ants. *Chem. Sci.* **2017**, *8*, 3218–3227, doi:10.1039/C6SC04265A.
56. Mevers, E.; Chouvenc, T.; Su, N.-Y.; Clardy, J. Chemical Interaction among Termite-Associated Microbes. *J. Chem. Ecol.* **2017**, *43*, 1078–1085, doi:10.1007/s10886-017-0900-6.
57. Shin, Y.-H.; Bae, S.; Sim, J.; Hur, J.; Jo, S.-I.; Shin, J.; Suh, Y.-G.; Oh, K.-B.; Oh, D.-C. Nicrophorusamides A and B, Antibacterial Chlorinated Cyclic Peptides from a Gut Bacterium of the Carrion Beetle *Nicrophorus Concolor*. *J. Nat. Prod.* **2017**, *80*, 2962–2968, doi:10.1021/acs.jnatprod.7b00506.
58. Beemelmanns, C.; Ramadhar, T.R.; Kim, K.H.; Klassen, J.L.; Cao, S.; Wyche, T.P.; Hou, Y.; Poulsen, M.; Bugni, T.S.; Currie, C.R.; et al. Macrotermycins A–D, Glycosylated Macrolactams from a Termite-Associated *Amycolatopsis* Sp. M39. *Org. Lett.* **2017**, *19*, 1000–1003, doi:10.1021/acs.orglett.6b03831.
59. Wyche, T.P.; Ruzzini, A.C.; Beemelmanns, C.; Kim, K.H.; Klassen, J.L.; Cao, S.; Poulsen, M.; Bugni, T.S.; Currie, C.R.; Clardy, J. Linear Peptides Are the Major Products of a Biosynthetic Pathway That Encodes for Cyclic Depsipeptides. *Org. Lett.* **2017**, *19*, 1772–1775, doi:10.1021/acs.orglett.7b00545.
60. Ma, S.Y.; Xiao, Y.S.; Zhang, B.; Shao, F.L.; Guo, Z.K.; Zhang, J.J.; Jiao, R.H.; Sun, Y.; Xu, Q.; Tan, R.X.; et al. Amycolamycins A and B, Two Enediyne-Derived Compounds from a Locust-Associated Actinomycete. *Org. Lett.* **2017**, *19*, 6208–6211, doi:10.1021/acs.orglett.7b03113.
61. Xiao, Y.S.; Zhang, B.; Zhang, M.; Guo, Z.K.; Deng, X.Z.; Shi, J.; Li, W.; Jiao, R.H.; Tan, R.X.; Ge, H.M. Rifamorpholines A–E, Potential Antibiotics from Locust-Associated Actinobacteria Amycolatopsis Sp. Hca4. *Org. Biomol. Chem.* **2017**, *15*, 3909–3916, doi:10.1039/C7OB00614D.

62. Human, Z.R.; Slippers, B.; De Beer, Z.W.; Wingfield, M.J.; Venter, S.N. Antifungal Actinomycetes Associated with the Pine Bark Beetle, *Orthotomicus Erosus*, in South Africa. *S. Afr. J. Sci.* **2017**, *Volume 113*, doi:10.17159/sajs.2017/20160215.
63. Arango, R.A.; Carlson, C.M.; Currie, C.R.; McDonald, B.R.; Book, A.J.; Green, F.; Lebow, N.K.; Raffa, K.F. Antimicrobial Activity of Actinobacteria Isolated From the Guts of Subterranean Termites. *Environ. Entomol.* **2016**, *45*, 1415–1423, doi:10.1093/ee/nvw126.
64. Axenov-Gribanov, D.; Rebets, Y.; Tokovenko, B.; Voytsekhovskaya, I.; Timofeyev, M.; Luzhetskyy, A. The Isolation and Characterization of Actinobacteria from Dominant Benthic Macroinvertebrates Endemic to Lake Baikal. *Folia Microbiol.* **2016**, *61*, 159–168, doi:10.1007/s12223-015-0421-z.
65. Holmes, N.A.; Innocent, T.M.; Heine, D.; Bassam, M.A.; Worsley, S.F.; Trottmann, F.; Patrick, E.H.; Yu, D.W.; Murrell, J.C.; Schiøtt, M.; et al. Genome Analysis of Two *Pseudonocardia* Phylotypes Associated with *Acromyrmex* Leafcutter Ants Reveals Their Biosynthetic Potential. *Front. Microbiol.* **2016**, *7*, doi:10.3389/fmicb.2016.02073.
66. Liu, S.; Xu, M.; Zhang, H.; Qi, H.; Zhang, J.; Liu, C.; Wang, J.; Xiang, W.; Wang, X. New Cytotoxic Spectinabilin Derivative from Ant-Associated *Streptomyces* Sp. 1H-GS5. *J. Antibiot.* **2016**, *69*, 128–131, doi:10.1038/ja.2015.99.
67. Liu, C.-X.; Liu, S.-H.; Zhao, J.-W.; Zhang, J.; Wang, X.-J.; Li, J.-S.; Wang, J.-D.; Xiang, W.-S. A New Spectinabilin Derivative with Cytotoxic Activity from Ant-Derived *Streptomyces* Sp. 1H-GS5. *J. As. Nat. Prod. Res.* **2017**, *19*, 924–929, doi:10.1080/10286020.2016.1254200.
68. Kang, H.R.; Lee, D.; Benndorf, R.; Jung, W.H.; Beemelmanns, C.; Kang, K.S.; Kim, K.H. Termisoflavones A–C, Isoflavonoid Glycosides from Termite-Associated *Streptomyces* Sp. RB1. *J. Nat. Prod.* **2016**, *79*, 3072–3078, doi:10.1021/acs.jnatprod.6b00738.
69. Um, S.; Bach, D.-H.; Shin, B.; Ahn, C.-H.; Kim, S.-H.; Bang, H.-S.; Oh, K.-B.; Lee, S.K.; Shin, J.; Oh, D.-C. Naphthoquinone–Oxindole Alkaloids, Coprisidins A and B, from a Gut-Associated Bacterium in the Dung Beetle, *Copris Tripartitus*. *Org. Lett.* **2016**, *18*, 5792–5795, doi:10.1021/acs.orglett.6b02555.
70. Van Arnam, E.B.; Ruzzini, A.C.; Sit, C.S.; Horn, H.; Pinto-Tomás, A.A.; Currie, C.R.; Clardy, J. Selvamicin, an Atypical Antifungal Polyene from Two Alternative Genomic Contexts. *Proc. Natl. Acad. Sci. U.S.A.* **2016**, *113*, 12940–12945, doi:10.1073/pnas.1613285113.
71. Bai, L.; Liu, C.; Guo, L.; Piao, C.; Li, Z.; Li, J.; Jia, F.; Wang, X.; Xiang, W. *Streptomyces Formicae* Sp. Nov., a Novel Actinomycete Isolated from the Head of *Camponotus Japonicus* Mayr. *Antonie Leeuwenhoek* **2016**, *109*, 253–261, doi:10.1007/s10482-015-0628-7.
72. Atlas, R.M. *Handbook of Microbiological Media*; 0 ed.; CRC Press, 2010; ISBN 978-0-429-13049-6.
73. Hsu, S.C.; Lockwood, J.L. Powdered Chitin Agar as a Selective Medium for Enumeration of Actinomycetes in Water and Soil. *Appl Microbiol* **1975**, *29*, 422–426, doi:10.1128/am.29.3.422-426.1975.
74. Hayakawa, M.; Nonomura, H. Humic Acid-Vitamin Agar, a New Medium for the Selective Isolation of Soil Actinomycetes. *J. Ferment. Technol.* **1987**, *65*, 501–509, doi:10.1016/0385-6380(87)90108-7.
75. Shirling, E.B.; Gottlieb, D. Methods for Characterization of *Streptomyces* Species. *Int. J. Syst. Bacteriol.* **1966**, *16*, 313–340, doi:10.1099/00207713-16-3-313.
76. Zhang, L.; Song, T.; Wu, J.; Zhang, S.; Yin, C.; Huang, F.; Hang, Y.; Abbas, N.; Liu, X.; Zhang, Y. Antibacterial and Cytotoxic Metabolites of Termite-Associated *Streptomyces* Sp. BYF63. *J Antibiot* **2020**, *73*, 766–771, doi:10.1038/s41429-020-0334-1.
77. Visser, A.A.; Nobre, T.; Currie, C.R.; Aanen, D.K.; Poulsen, M. Exploring the Potential for Actinobacteria as Defensive Symbionts in Fungus-Growing Termites. *Microb Ecol* **2012**, *63*, 975–985, doi:10.1007/s00248-011-9987-4.
78. Du, Y.E.; Byun, W.S.; Lee, S.B.; Hwang, S.; Shin, Y.-H.; Shin, B.; Jang, Y.-J.; Hong, S.; Shin, J.; Lee, S.K.; et al. Formicins, *N*-Acetylcysteamine-Bearing Indenone Thioesters from a Wood Ant-Associated Bacterium. *Org. Lett.* **2020**, *22*, 5337–5341, doi:10.1021/acs.orglett.0c01584.
79. *Practical Streptomyces Genetics*; Kieser, T., Ed.; Innes: Norwich, 2000; ISBN 978-0-7084-0623-6.

Back to contents

80. Shin, Y.-H.; Bae, S.; Sim, J.; Hur, J.; Jo, S.-I.; Shin, J.; Suh, Y.-G.; Oh, K.-B.; Oh, D.-C. Nicrophorusamides A and B, Antibacterial Chlorinated Cyclic Peptides from a Gut Bacterium of the Carrion Beetle *Nicrophorus Concolor*. *J. Nat. Prod.* **2017**, *80*, 2962–2968, doi:10.1021/acs.jnatprod.7b00506.
81. Tan, G.Y.A.; Ward, A.C.; Goodfellow, M. Exploration of *Amycolatopsis* Diversity in Soil Using Genus-Specific Primers and Novel Selective Media. *Systematic and Applied Microbiology* **2006**, *29*, 557–569, doi:10.1016/j.syapm.2006.01.007.
82. Chouvenc, T.; Efstathion, C.A.; Elliott, M.L.; Su, N.-Y. Extended Disease Resistance Emerging from the Faecal Nest of a Subterranean Termite. *Proc. R. Soc. B.* **2013**, *280*, 20131885, doi:10.1098/rspb.2013.1885.
83. Qin, S.; Chen, H.-H.; Zhao, G.-Z.; Li, J.; Zhu, W.-Y.; Xu, L.-H.; Jiang, J.-H.; Li, W.-J. Abundant and Diverse Endophytic Actinobacteria Associated with Medicinal Plant *Maytenus Austroyunnanensis* in Xishuangbanna Tropical Rainforest Revealed by Culture-Dependent and Culture-Independent Methods: Endophytic Actinobacteria from *Maytenus Austroyunnanensis*. *Environ. Microbiol. Rep.* **2012**, *4*, 522–531, doi:10.1111/j.1758-2229.2012.00357.x.

List S2. Excluded publications

1. Park, S.-H.; Moon, K.; Bang, H.-S.; Kim, S.-H.; Kim, D.-G.; Oh, K.-B.; Shin, J.; Oh, D.-C. Tripartilactam, a Cyclobutane-Bearing Tricyclic Lactam from a *Streptomyces* Sp. in a Dung Beetle's Brood Ball. *Org. Lett.* **2012**, *14*, 1258–1261, doi:10.1021/ol300108z. (source - dung beetle's brood ball)
2. Madden, A.A.; Grassetti, A.; Soriano, J.-A.N.; Starks, P.T. Actinomycetes with Antimicrobial Activity Isolated from Paper Wasp (Hymenoptera: Vespidae: Polistinae) Nests. *Environ. Entomol.* **2013**, *42*, 703–710, doi:10.1603/EN12159. (source - paper wasp nest)
3. Kim, K.H.; Ramadhar, T.R.; Beemelmanns, C.; Cao, S.; Poulsen, M.; Currie, C.R.; Clardy, J. Natalamycin A, an Ansamycin from a Termite-Associated Streptomyces Sp. *Chem. Sci.* **2014**, *5*, 4333–4338, doi:10.1039/C4SC01136H. (source - termite comb)
4. Sujada, N.; Sungthong, R.; Lumyong, S. Termite Nests as an Abundant Source of Cultivable Actinobacteria for Biotechnological Purposes. *Microb. Environ.* **2014**, *29*, 211–219, doi:10.1264/jsme2.ME13183. (source - termite nests)
5. Padilla, M.A.; Rodrigues, R.A.F.; Bastos, J.C.S.; Martini, M.C.; Barnabé, A.C. de S.; Kohn, L.K.; Uetanabaro, A.P.T.; Bomfim, G.F.; Afonso, R.S.; Fantinatti-Garboggini, F.; et al. Actinobacteria from Termite Mounds Show Antiviral Activity against Bovine Viral Diarrhea Virus, a Surrogate Model for Hepatitis C Virus. *Evidence-Based Complem. Altern. Med.* **2015**, *2015*, 1–9, doi:10.1155/2015/745754. (source - termite mounds)
6. Romero, C.A.; Grkovic, T.; Han, J.; Zhang, L.; French, J.R.J.; Kurtböke, D.I.; Quinn, R.J. NMR Fingerprints, an Integrated Approach to Uncover the Unique Components of the Drug-like Natural Product Metabolome of Termite Gut-Associated Streptomyces Species. *RSC Adv.* **2015**, *5*, 104524–104534, doi:10.1039/C5RA17553D. (source - termite comb)
7. Krishanti, Ni.; Antimicrobial Production by an Actinomycetes Isolated from The Termite Nest. *J. Trop. Life Sci.* **2018**, *8*, 279–288, doi:10.11594/jtls.08.03.10. (source - termite nests)
8. Klassen, J.L.; Lee, S.R.; Poulsen, M.; Beemelmanns, C.; Kim, K.H. Efomycins K and L From a Termite-Associated Streptomyces Sp. M56 and Their Putative Biosynthetic Origin. *Front. Microbiol.* **2019**, *10*, 1739, doi:10.3389/fmicb.2019.01739. (source - termite fungus comb)
9. Yin, C.; Jin, L.; Li, S.; Xu, X.; Zhang, Y. Diversity and Antagonistic Potential of Actinobacteria from the Fungus-Growing Termite Odontotermes Formosanus. *3 Biotech* **2019**, *9*, 45, doi:10.1007/s13205-019-1573-3. (source - .termite nest)
10. Efimenko, T.A.; Glukhova, A.A.; Demiankova, M.V.; Boykova, Y.V.; Malkina, N.D.; Sumarukova, I.G.; Vasilieva, B.F.; Rogozhin, E.A.; Ivanov, I.A.; Krassilnikov, V.A.; et al. Antimicrobial Activity of Microorganisms Isolated from Ant Nests of Lasius Niger. *Life* **2020**, *10*, 91, doi:10.3390/life10060091. (source - ant nests)
11. Hwang, S.; Kim, E.; Lee, J.; Shin, J.; Yoon, Y.J.; Oh, D.-C. Structure Revision and the Biosynthetic Pathway of Tripartilactam. *J. Nat. Prod.* **2020**, *83*, 578–583, doi:10.1021/acs.jnatprod.9b00819. (source - dung beetle's brood ball)
12. Lee, S.R.; Lee, D.; Park, M.; Lee, J.C.; Park, H.-J.; Kang, K.S.; Kim, C.-E.; Beemelmanns, C.; Kim, K.H. Absolute Configuration and Corrected NMR Assignment of 17-Hydroxycyclooctatin, a Fused 5–8–5 Tricyclic Diterpene. *J. Nat. Prod.* **2020**, *83*, 354–361, doi:10.1021/acs.jnatprod.9b00837. (source - termite comb)

13. Grubbs, K.J.; May, D.S.; Sardina, J.A.; Dermenjian, R.K.; Wyche, T.P.; Pinto-Tomás, A.A.; Clardy, J.; Currie, C.R. Pollen Streptomyces Produce Antibiotic That Inhibits the Honey Bee Pathogen Paenibacillus Larvae. *Front. Microbiol.* **2021**, *12*, 632637, doi:10.3389/fmicb.2021.632637. (source - bee pollen)

14. Long, Y.; Zhang, Y.; Huang, F.; Liu, S.; Gao, T.; Zhang, Y. Diversity and Antimicrobial Activities of Culturable Actinomycetes from Odontotermes Formosanus (Blattaria: Termitidae). *BMC Microbiol.* **2022**, *22*, 80, doi:10.1186/s12866-022-02501-5. (source - termite comb)

Back to contents
